# Genome-guided characterization of *Weissella* species highlights strain-specific functional traits relevant to food fermentation and probiotics

**DOI:** 10.1128/spectrum.00331-26

**Published:** 2026-07-13

**Authors:** Nattarika Chaichana, Kamonnut Singkhamanan, Monwadee Wonglapsuwan, Rattanaruji Pomwised, Komwit Surachat

**Affiliations:** 1 Department of Biomedical Sciences and Biomedical Engineering, Faculty of Medicine, Prince of Songkla University 26686, Hat Yai, Songkhla, Thailand; 2 Division of Biological Science, Faculty of Science, Prince of Songkla University 26686, Hat Yai, Songkhla, Thailand; University of Valencia, Paterna, Valencia, Spain

**Keywords:** *Weissella*, comparative genomics, pan-genome analysis, exopolysaccharide biosynthesis, probiotic potential

## Abstract

The genus *Weissella* comprises heterofermentative lactic acid bacteria (LAB) widely distributed in fermented foods, plant-associated environments, and host-associated niches. However, the functional diversity, safety-related genomic features, and evolutionary dynamics of the genus remain incompletely understood at the genomic level. This study performed a comprehensive comparative genomic analysis of 347 high-quality *Weissella* genomes representing 16 assigned species to elucidate their genomic diversity, pan-genome architecture, and functional potential relevant to food fermentation, probiotics, and biotechnology. Pan-genome analysis revealed an open pan-genome following a power-law model with an exponent of 0.304, dominated by shell and cloud genes, indicating substantial genomic plasticity and ongoing gene acquisition. *In silico* safety screening showed that the vast majority of genomes lacked detectable antimicrobial resistance or virulence-associated genes under the applied criteria, indicating a low detectable burden of these genetic determinants across the data set. Moreover, functional annotation revealed conserved carbohydrate-active enzyme (CAZyme) repertoires dominated by glycoside hydrolases (GHs) and glycosyltransferases (GTs), alongside species-specific variation in carbohydrate-binding modules (CBMs). Comparative synteny analysis identified six distinct architectures of exopolysaccharide (EPS) biosynthesis loci, with *W. cibaria* more frequently harboring regulator-rich, putatively complete EPS gene clusters. Putative probiotic-associated genetic markers were unevenly distributed across species, mainly involving stress resistance, vitamin biosynthesis, GIT tolerance, and oxidative stress resistance, whereas GABA production and antimicrobial metabolite-associated markers were not detected in the data set. Hence, this genome-resolved analysis provides a systematic framework for understanding *Weissella* diversity and supports a genome-guided, strain-level approach for prioritizing candidate strains for future experimental validation in food, health, and biotechnological applications.

**IMPORTANCE:**

Microbial traits relevant to food fermentation, probiotic performance, and safety emerge from complex interactions among core metabolism, accessory genes, and genome plasticity; however, these relationships remain poorly resolved at the genus scale. By analyzing 347 high-quality genomes spanning 16 *Weissella* species, this study reveals how an open pan-genome, extensive accessory gene diversity, and lineage-specific gene architectures shape functional potential across this lactic acid bacterial genus. Key traits, including EPS biosynthesis, carbohydrate utilization capacity, stress resilience, and biosynthetic gene cluster distribution, are unevenly structured across species and strains, emphasizing the need for genome-guided strain selection rather than taxonomic assumptions. Integrating phylogenomics, gene content variation, and functional profiling links genome evolution with applied phenotypes. These findings advance understanding of how genomic diversity underpins ecological adaptation and biotechnological performance and provide a blueprint for selecting safe and functionally optimized strains in food and health applications.

## INTRODUCTION

The genus *Weissella*, belonging to the family Leuconostocaceae, consists of gram-positive, catalase-negative, and heterofermentative lactic acid bacteria (LAB). Cells appear as cocci or short rods, typically arranged in pairs or small chains (1). These microorganisms are widespread in nature and are frequently recovered from fermented foods, plant materials, and the gastrointestinal or oral microbiota of humans and animals, where they contribute to the fermentation of carbohydrates and the production of lactic acid (2, 3). Advances in phylogenetic and genomic studies have refined the taxonomy of *Weissella*. Recent reclassifications clarified species boundaries and introduced the related genus *Periweissella*, providing greater resolution for ecological and biotechnological research within the Leuconostocaceae family. These taxonomic updates are crucial because species identity often determines functional roles, including exopolysaccharide synthesis and probiotic potential, as well as safety considerations (4). From a technological perspective, *Weissella* species are particularly valued in food fermentation processes. Certain strains are prolific producers of exopolysaccharides (EPS), such as dextran, which can improve the texture, water-binding capacity, and sensory attributes of bread, vegetable ferments, and other products (5, 6). In addition, several strains synthesize bacteriocins, notably weissellicins, that display antimicrobial activity against a variety of spoilage and pathogenic bacteria, making them attractive as natural food biopreservatives (7, 8). The health-promoting potential of *Weissella* has been investigated most extensively in *W. cibaria* (9). Clinical trials, such as a randomized controlled study of *W. cibaria* strain CMU, have demonstrated improvements in periodontal health and modulation of oral microbiota (10). Laboratory studies of *W. cibaria* strains further support its antimicrobial, anti-biofilm, and immunomodulatory properties against various pathogens, including *Streptococcus pyogenes*, *Staphylococcus aureus*, *S. pneumoniae*, *Moraxella catarrhalis,* and *S. mutans* (11–13). Although promising, these benefits are strongly strain-dependent, and thorough safety evaluations remain essential, particularly as the genus’s broad metabolic capacity can have significant implications for both industrial fermentation processes and human health applications. In this context, our study provides an in-depth comparative genomic analysis of 347 *Weissella* strains, highlighting the functional diversity of these bacteria and offering new insights into their evolutionary trajectories, probiotic potential, and suitability for industrial applications. By integrating genomic perspectives, this research lays the foundation for the rational selection of *Weissella* strains for biotechnological use, emphasizing strain-level validation for safety and functionality.

## MATERIALS AND METHODS

### Data collection, quality control, and average amino acid identity (AAI) analysis

For the data collection of *Weissella* genomes available in the NCBI database, the data retrieval and quality control steps include:

All 388 *Weissella* genomes were downloaded from the NCBI database (accessed November 3, 2025).

Quality control (QC) was done to get good-quality genomes by excluding atypical genomes and metagenome-assembled genomes (MAGs). The genomes with >200 contigs were also excluded from the analysis. The quality was assessed by CheckM (14), retaining only genomes with estimated completeness ≥95% and contamination ≤5% and Busco completeness ≥95% (15).

Finally, a total of 347 high-quality genomes among the *Weissella* genus were used for further downstream analysis.

Moreover, all genomes were re-annotated to their taxonomy using the Genome Taxonomy Database Toolkit (GTDB-Tk) (16) and analyzed for average amino acid identity (AAI) among them (17).

### Genome annotation

All the genomes were annotated using the Prokka software using default parameters (18). The output of the Prokka annotation was used for other identifications.

### Phylogenetic analysis and comparative genomics

The pan-genome analysis of all 347 *Weissella* strains was constructed using Panaroo v1.5.2 (19), with clean-mode strict parameters. After running Panaroo, the pan-genome was divided into the core (*>*99%), soft core (*<*99% to ≥95%), shell (*<*95% to ≥15%), and cloud (*<*15%) genomes. Pan-genome openness was assessed using Heap’s law based on the Panaroo gene presence-absence matrix, with accumulation curves generated from 1,000 random genome permutations and fitted to the model (P = kN^λ^) using log-transformed linear regression, where P is the pan-genome size, N is the number of genomes, and k and λ are fitting parameters. The parameter λ was used to assess the openness of the pan-genome. Under this formulation, positive values of λ indicate an open pan-genome that continues to expand as additional genomes are sampled, whereas values approaching zero suggest slower expansion and a tendency toward saturation. Larger λ values reflect faster rates of new gene accumulation. In addition, a maximum-likelihood phylogenetic tree was generated using FastTree v2.1 (20) and subsequently annotated and visualized using the Interactive Tree of Life (iTOL) v8.

### *In silico* safety analysis

Genes potentially linked to safety risks were also predicted. Antimicrobial resistance genes (ARGs) were identified using AMRFinderPlus (NCBI) (21) on both the assembled contigs and the Prokka-annotated protein sequences. To independently validate the presence of acquired AMR genes, contigs were additionally analyzed with ABRicate against the ResFinder database, retaining only matches with ≥50% coverage and ≥90% identity (22). Putative virulence factors were screened using ABRicate with the VFDB database (23), applying more stringent criteria (≥70% coverage and ≥90% identity) to reduce false positives in lactic acid bacteria. ABRicate was executed with default settings following the database setup.

### Genotype prediction of carbohydrate-active enzymes

The genes for the enzymes of carbohydrates in the *Weissella* genomes were identified using dbCAN3 with the DIAMOND tool against the CAZy database (24). The database primarily categorizes enzymes into six major groups, including glycoside hydrolases (GHs), glycosyltransferases (GTs), carbohydrate esterases (CEs), carbohydrate-binding modules (CBMs), auxiliary activity enzymes (AAs), and polysaccharide lyases (PLs).

### Comparative analysis of EPS gene clusters across genomes

EPS-related genes were identified in all 347 *Weissella* genomes using the dbCAN3 annotation pipeline (24), with protein sequences analyzed by HMMER to detect CAZyme hits, including GTs and other EPS-associated genes, such as Wzx/Wzy flippases and polymerases. These genes were mapped to their genomic locations using GenBank (.gbk) files, and genomic regions containing co-localized EPS-related genes were extracted as putative EPS biosynthesis clusters. These clusters were grouped into six synteny sub-groups based on their gene order and the presence of key EPS enzymes and regulators. The sub-groups reflect different conserved architectures of EPS loci. Sub-group A represents medium-sized loci containing insertion sequence (IS) elements. Sub-group B contains full-length loci with conserved gene order and core EPS genes. Sub-group C includes full-length loci enriched with sugar-modifying enzymes and regulatory genes. Sub-group D consists of full-length loci with abundant glycosyltransferases and regulators. Sub-group E includes full-length loci with a high abundance of insertion sequences and transposases, whereas sub-group F represents compact loci with a high proportion of hypothetical proteins and relatively few EPS-related enzymes. These cluster-specific GenBank files were compared using clinker (25), which generated gene-level homology maps and synteny visualizations for comparative analysis across species.

### Biosynthetic gene cluster (BGC) analysis

The BGCs associated with secondary metabolites across 347 *Weissella* genomes were detected using antiSMASH v8.0 (26), while BAGEL4 was applied to predict gene clusters related to bacteriocins (27).

### Probiotic and techno-functional genes

Probiotic-associated genes related to gastrointestinal (GIT) tolerance, adhesion, BGC-derived metabolite production, the γ-aminobutyric acid (GABA) system, stress resistance, vitamin biosynthesis, and oxidative stress defense were identified across isolates and linked to species metadata. Gene prevalence was subsequently calculated based on Panaroo-derived gene presence/absence matrices.

### Data visualization and statistical analysis

All information on species-level functional profiles was summarized, and visualization was performed using Python v3.13.7. To evaluate whether EPS synteny sub-groups were over-represented in particular species, each species/sub-group combination was compared against all remaining EPS clusters using one-sided Fisher’s exact tests. *P* values were adjusted for multiple comparisons using the Benjamini-Hochberg false discovery rate method, with *q* < 0.05 considered statistically significant. Formal enrichment testing was restricted to analyses with explicit count data; normalized probiotic-associated functional profiles were interpreted descriptively rather than as statistically tested enrichment.

## RESULTS

### Genome distribution among *Weissella* species

**Fig 1:**
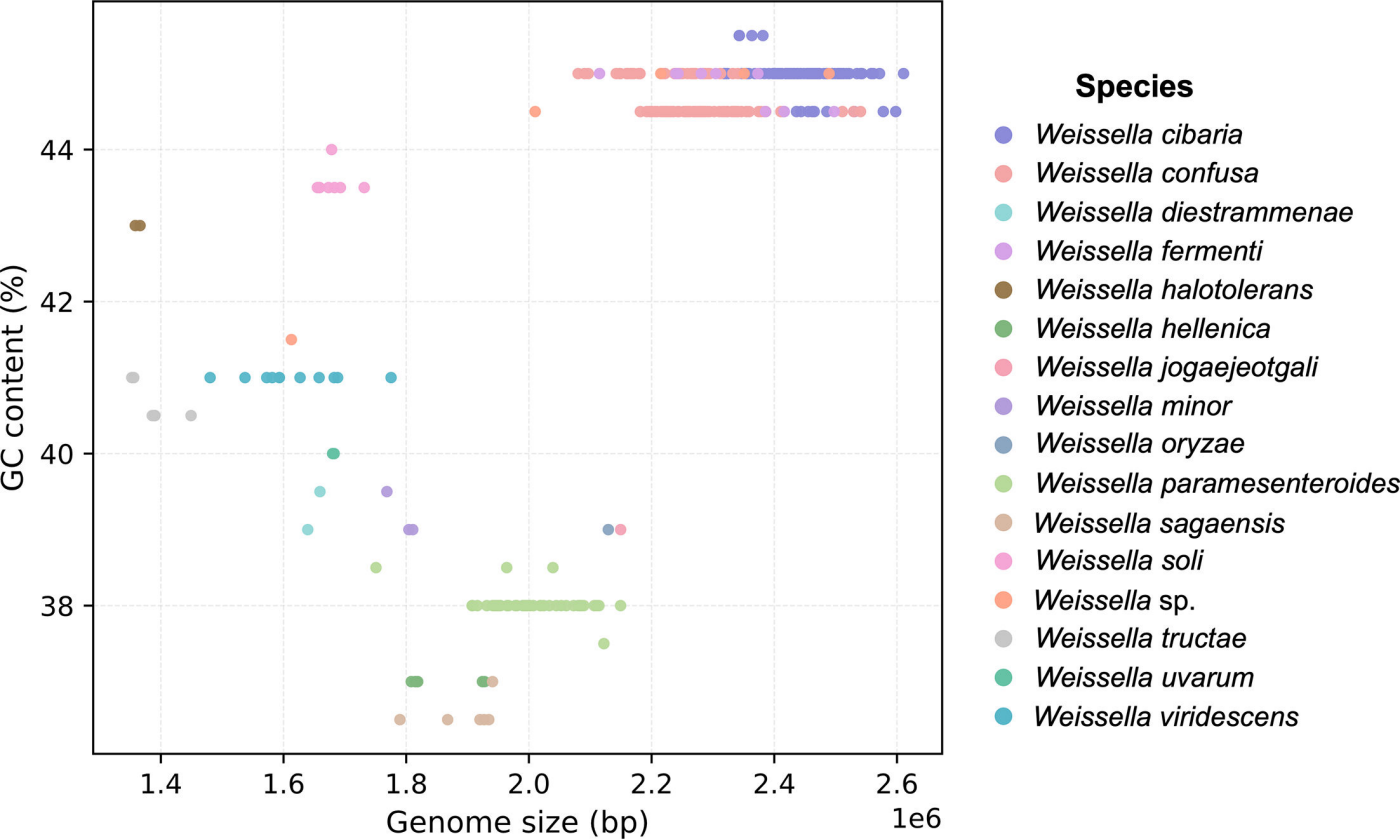
Genome size and GC content of *Weissella* genomes from the NCBI data set. Scatter plot of 347 *Weissella* genomes showing species-specific clustering patterns across genome size (1.35–2.61 Mbp) and GC content (36.58%–45.50%). Each dot represents one genome, colored by species.

A total of 388 *Weissella* genomes representing all assembly levels were retrieved from the NCBI RefSeq database on November 3, 2025. To ensure a high-quality data set for downstream comparative analyses, genome assemblies were curated to confirm accurate taxonomic assignment, assembly quality, and consistent annotation. Genomes classified as atypical assemblies or MAGs, as well as those containing more than 200 contigs or failing basic QC criteria, were excluded. After removing 41 low-quality genomes, a total of 347 high-quality assemblies representing 15 *Weissella* named species from NCBI were retained for further analysis (Table S1). The relationship between genome size and GC content across all genomes is illustrated in Fig. 1, where each species forms a distinct cluster based on its genomic characteristics. The genome sizes of the analyzed *Weissella* species ranged from 1.35 Mbp to 2.61 Mbp, with an average genome length of 2.20 Mbp. The smallest genomes were identified in *W. tructae* (1.35 Mbp), while the largest genome was observed in *W. cibaria* (2.61 Mbp). The GC content varied from 36.58% to 45.50%, with a mean GC content of 41.35% across all genomes. The lowest GC content was found in *W. sagaensis* (36.58%), whereas the highest values were recorded in *W. cibaria* (45.50%). The species distribution within the data set was dominated by *W. confusa* (*n* = 119) and *W. cibaria* (*n* = 109), followed by *W. paramesenteroides* (*n* = 56). Moderate representation was observed for *W. viridescens* (*n* = 11), *W. fermenti* (*n* = 9), *W. soli* (*n* = 8), *W. hellenica* (*n* = 7), *W. tructae* (*n* = 6), and *W. sagaensis* (*n* = 6). The remaining species were represented by fewer than five genomes each (Table S2).

### AAI analysis

**Fig 2:**
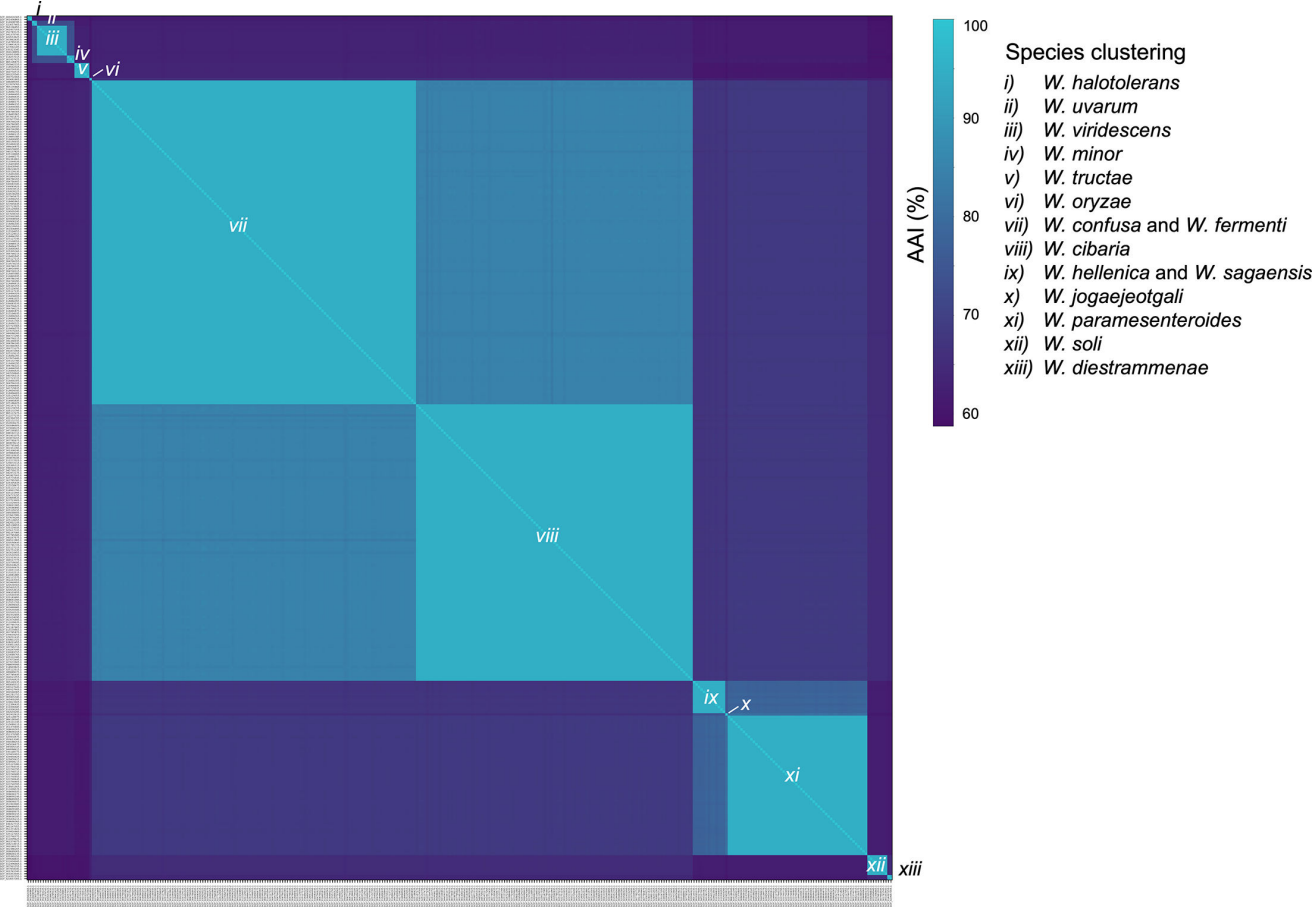
The AAI heatmap of 347 *Weissella* genomes. Heatmap showing pairwise AAI values among 347 *Weissella* genomes. Distinct clusters correspond to major species groups, with clear separation between species. Smaller blocks represent species with fewer genomes, and the lower AAI values between species are visible outside the diagonal clusters.

AAI analysis of the 347 high-quality *Weissella* genomes revealed clear genome-wide species boundaries and strong taxonomic coherence across the genus (Fig. 2). The pairwise AAI heatmap showed multiple well-defined diagonal clusters of high within-group similarity, indicating that genomes assigned to the same species formed cohesive genomic groups with limited overlap between species. This pattern supports the robustness of current species assignments and demonstrates the utility of AAI for resolving interspecies relationships within *Weissella*. The two largest and most continuous clusters corresponded to *W. confusa* and *W. cibaria*, reflecting both their high genomic relatedness within species and their dominant representation in the data set. *W. paramesenteroides* formed a distinct and cohesive cluster of moderate size. In contrast, less frequently represented species, including *W. halotolerans*, *W. uvarum*, *W. viridescens*, *W. minor, W. tructae, W. oryzae, W. jogaejeotgali*, *W. soli*, and *W. diestrammenae*, appeared as smaller but clearly separated high-identity blocks, consistent with reduced sampling depth rather than weak species coherence. Notably, two closely related species pairs, *W. confusa* and *W. fermenti*, and *W. hellenica* with *W. sagaensis*, formed adjacent clustering patterns with elevated intergroup similarity relative to other species comparisons. This suggests closer evolutionary relationships between these taxa while still maintaining separable species-level boundaries. The sharp contrast between high within-species AAI and lower between-species similarity indicates pronounced genomic differentiation across the genus and provides a robust framework for downstream comparative analyses.

### Pan-genome analysis and phylogenomic tree construction

**Fig 3:**
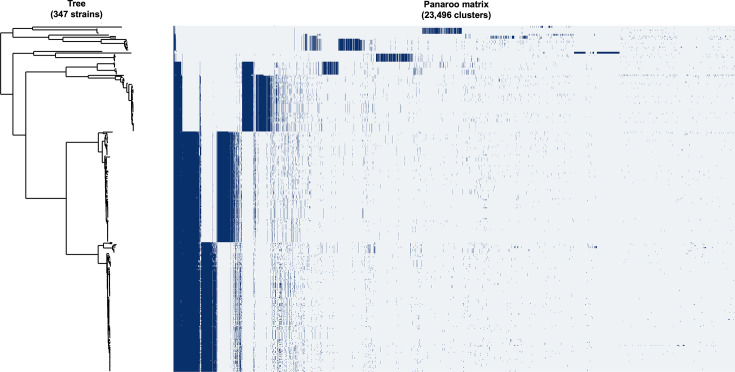
Pan-genome analysis of *Weissella* strains. The pan-genome matrix shows the presence or absence of 23,496 gene clusters across 347 *Weissella* strains, categorized into core genes (29 genes, 99%–100% present), soft core genes (40 genes, 95%–99%), shell genes (4,025 genes, 15%–95%), and cloud genes (19,402 genes, 0%–15%). The hierarchical tree on the left represents strain clustering based on genetic similarity, illustrating the high genomic diversity within the genus.

The pan-genome analysis of 347 *Weissella* strains revealed significant genomic diversity, as illustrated by Fig. 3. A total of 23,496 gene clusters were identified, which were categorized into core, soft core, shell, and cloud genes based on their distribution across the strains. The core genes (29 genes) are present in 99% to 100% of the strains, while the soft core genes (40 genes) are present in 95%–99% of the strains. The shell genes (4,025 genes) were found in 15% to <95% of the strains, while the cloud genes (19,402 genes) were present in less than 15% of the strains (Fig. S1). Moreover, Heap’s law fitting of the pan-genome accumulation curve followed a power-law model with an exponent of 0.304, indicating that the *Weissella* pan-genome remains open and continues to expand as additional genomes are sampled, although the rate of new gene acquisition is relatively gradual.

**Fig 4:**
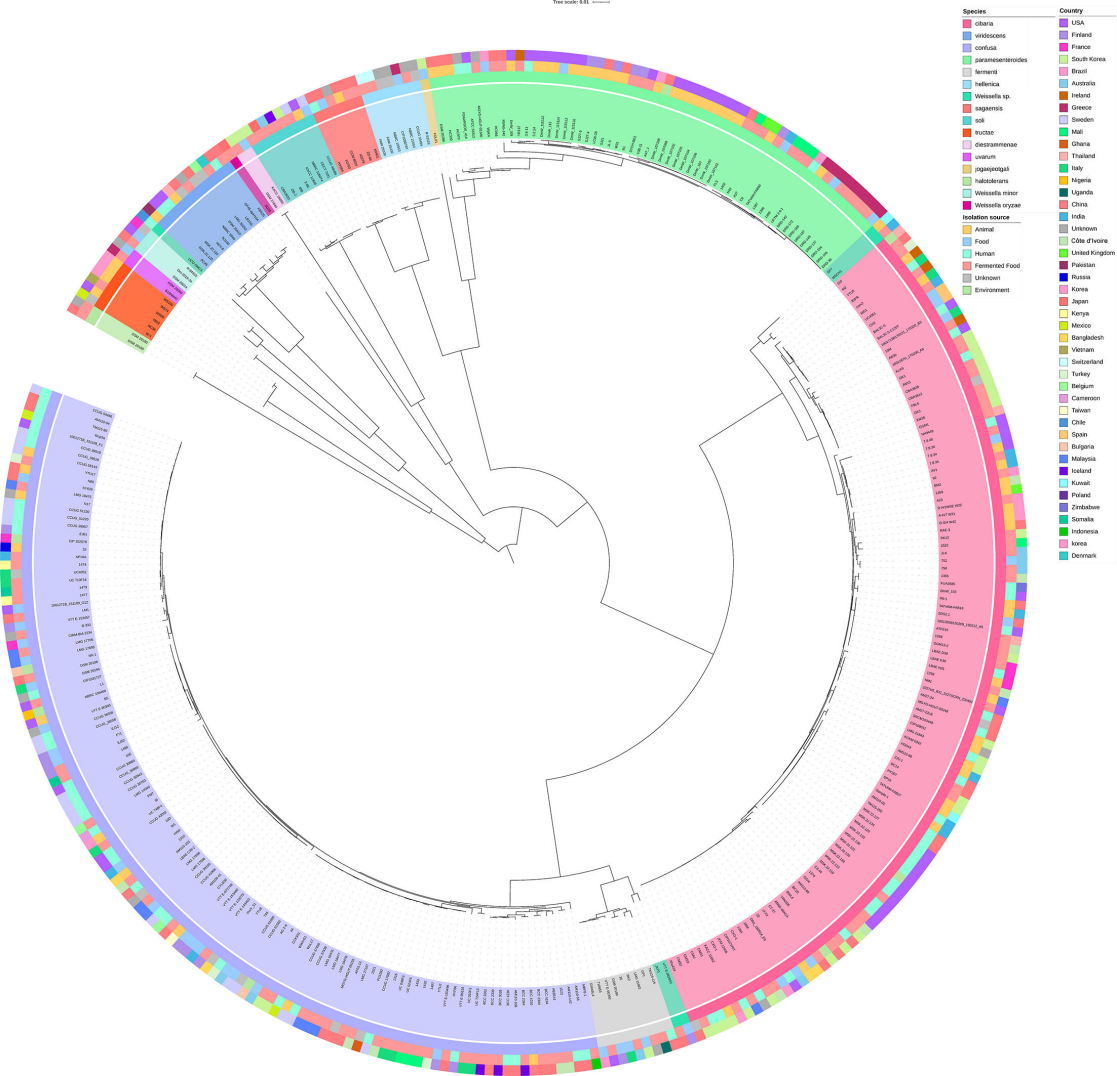
Core genome phylogeny of 347 *Weissella* genomes. The circular tree was reconstructed from the Panaroo core gene alignment using FastTree. Colored rings indicate species, isolation source, and country of origin. Genomes annotated as *Weissella* sp. formed a distinct lineage, suggesting potential unresolved taxonomic diversity.

In addition, a phylogenomic tree reconstructed from the Panaroo core gene alignment revealed a strong species-level population structure across the 347 *Weissella* genomes, indicating that conserved genomic regions retain clear evolutionary signals throughout the genus (Fig. 4). The largest and most densely populated clades corresponded to *W. confusa* and *W. cibaria*, consistent with their numerical dominance in the data set. These species formed well-supported monophyletic groups with substantial internal branching, suggesting notable intraspecies diversity despite overall genomic cohesion. Also, *W. paramesenteroides* also formed a distinct and compact lineage, clearly separated from the two dominant clades. Several moderately represented species, including *W. viridescens, W. soli, W. hellenica*, and *W. sagaensis*, formed discrete monophyletic branches, supporting their current taxonomic assignments. Species represented by fewer genomes, such as *W. oryzae, W. fermenti, W. jogaejeotgali, W. uvarum*, and *W. minor*, appeared as short independent lineages, reflecting both lower sampling depth and narrower genomic representation. Closely related species pairs, particularly *W. confusa* with *W. fermenti* and *W. hellenica* with *W. sagaensis*, were positioned on neighboring branches, suggesting closer evolutionary relatedness relative to other members of the genus. Notably, genomes designated as *Weissella* sp. did not cluster within any established species clade but instead formed a separate lineage distinct from recognized taxa. Integration of metadata showed that ecological origin was broadly distributed across the phylogeny rather than strictly lineage-specific. Fermented food isolates represented the largest proportion of genomes and were widely distributed, particularly within the *W. confusa* and *W. cibaria* clades, reinforcing the ecological importance of these species in food fermentation systems. Animal-associated isolates were observed across multiple lineages, with stronger representation in *W. paramesenteroides, W. sagaensis,* and *W. tructae*. Human-derived, environmental, insect-associated, and raw food isolates were less frequent but scattered throughout the tree, indicating repeated adaptation of different *Weissella* lineages to diverse ecological niches. Notably, *W. confusa* showed the broadest source diversity, encompassing fermented food, raw food, environmental, animal, and human-associated origins, consistent with a generalist ecological profile. In contrast, *W. cibaria* remained predominantly associated with fermented foods, whereas several minor species displayed narrower source distributions. Geographic metadata further demonstrated broad international distribution across the genus. Major species, especially *W. confusa* and *W. cibaria*, contained strains originating from multiple countries and continents, reflecting both ecological success and intensive sampling. In contrast, sparsely represented taxa were often restricted to one or a few countries, likely reflecting limited genome availability rather than true endemism.

### *In silico* screening for antimicrobial resistance and virulence factor-associated genes among *Weissella* species

**Fig 5:**
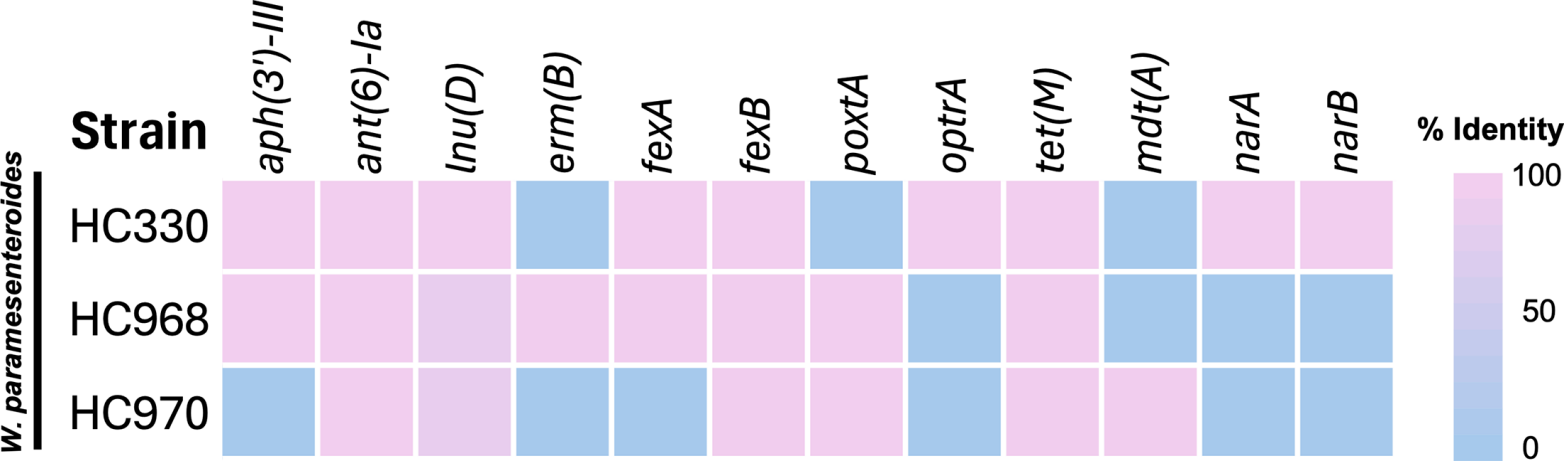
Distribution of ARGs among 347 *Weissella* genomes. Heatmap showing the presence of ARGs across all analyzed *Weissella* strains. Only three strains carried detectable ARGs, while the remaining 344 strains showed no ARGs in the database screen. Each column represents an individual resistance gene, and each row corresponds to a strain, with colored cells indicating gene presence.

Screening of 347 *Weissella* genomes showed that acquired ARGs were rare across the data set, being detected in only three strains (HC330, HC968, and HC970), whereas the remaining 344 genomes contained no identifiable ARGs (Fig. 5). The detected ARGs represented multiple antibiotic classes, indicating that resistance in these strains was not limited to a single mechanism. Shared genes included *lnu(D*), detected in all three strains, and *ant(6)-Ia* and *tet(M*), which were also consistently present and associated with resistance to lincosamides, aminoglycosides, and tetracyclines, respectively. Additional genes, including *aph(3*′*)-III*, *fexA, poxtA, optrA*, and *erm(B*), were identified in a subset of strains, further expanding the predicted resistance spectrum to phenicols, oxazolidinones, and MLSB antibiotics. HC330 carried the broadest ARG repertoire, including *narA* and *narB*, whereas HC970 contained the narrowest set, with *mdt(A*) as its only strain-specific determinant. In contrast to the limited and strain-restricted ARG distribution, no virulence factor-associated genes were detected in any of the 347 genomes under the applied screening criteria. These results indicate that the *Weissella* genus exhibits a generally low genomic burden of acquired resistance determinants, with potential safety concerns confined to a very small number of individual strains rather than representing a genus-wide feature.

### CAZyme distribution among *Weissella* species

**Fig 6:**
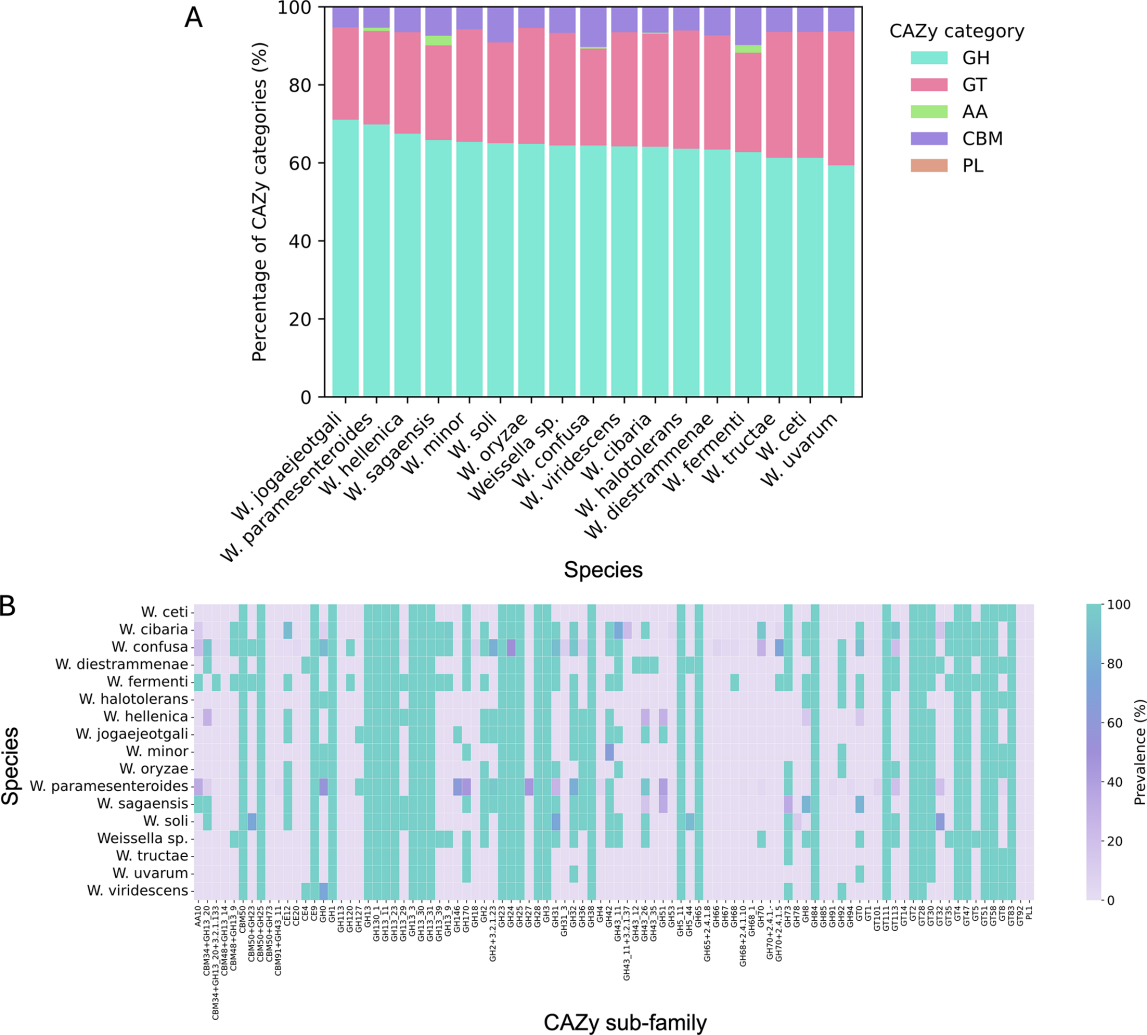
Relative distribution (%) of CAZy categories across *Weissella* species. (**A**) For each species, bars represent the proportional contribution (%) of GH, GT, AA, CBM, and PL, calculated from the mean number of CAZymes per genome, and colors indicate CAZy categories. (**B**) Heatmap showing the prevalence (%) of CAZy sub-families across *Weissella* species. Color intensity indicates the prevalence of each sub-family within a species.

The CAZyme analysis revealed a highly conserved distribution among *Weissella* species. In all species examined, GHs constituted the largest proportion of the CAZy repertoire, accounting for approximately 60%–70% of the total CAZymes per genome. This dominance was consistently observed across both highly represented species (*W. confusa*, *W. cibaria*, and *W. paramesenteroides*) and moderately represented species. GTs represented the second most abundant CAZy category, contributing roughly 20%–30% of the total CAZy content in each species. The relative proportion of GTs was stable across species, with only minor interspecific variation. However, CBMs were present at lower proportions compared with GHs and GTs but showed noticeable variation among species. In particular, *W. confusa* exhibited a higher relative CBM contribution than other species, whereas reduced CBM proportions were observed in *W. paramesenteroides* and several moderately represented species. In contrast, AAs and PLs together accounted for only a small fraction of the CAZy repertoire, typically less than 5% of the total CAZymes. These categories were sporadically distributed and absent in several species, indicating limited representation across the genus (Fig. 6A). Among these categories, the heatmap analysis of CAZy sub-family prevalence revealed a distinct yet structured distribution pattern across *Weissella* species (Fig. 6B). A set of core CAZyme sub-families showed consistently high prevalence across *Weissella* species, including GH1, GH13-related families (GH13, GH13_3, GH13_11, GH13_23, GH13_30, GH13_31, and GH130_1), GH23, GH24, GH25, GH28, GH4, GH47, GH51, GH58, GH8, together with GT2, GT4, GT8, GT28, GT47, GT51, GT58, GT111, and the carbohydrate-binding modules CBM50-related families. These conserved sub-families were present in both dominant species (*W. confusa, W. cibaria*, and *W. paramesenteroides*) and less represented taxa. In contrast, accessory CAZy sub-families exhibited marked species-specific variation. Several GH sub-families and CBM-associated modules displayed heterogeneous prevalence patterns, with higher representation in *W. confusa* and *W. cibaria* compared with other species. Moreover, *W. paramesenteroides* showed an intermediate distribution pattern, retaining the conserved core sub-families but exhibiting reduced prevalence of accessory components. Moderately represented species, including *W. viridescens, W. soli, W. hellenica*, and *W. sagaensis*, generally showed narrower CAZy sub-family profiles, characterized by the presence of core GH sub-families but limited diversity of accessory sub-families. Several CAZy sub-families were detected only sporadically or at low prevalence, indicating restricted distribution among specific species.

### Distribution of putative probiotic-associated genetic potential across *Weissella* species

**Fig 7:**
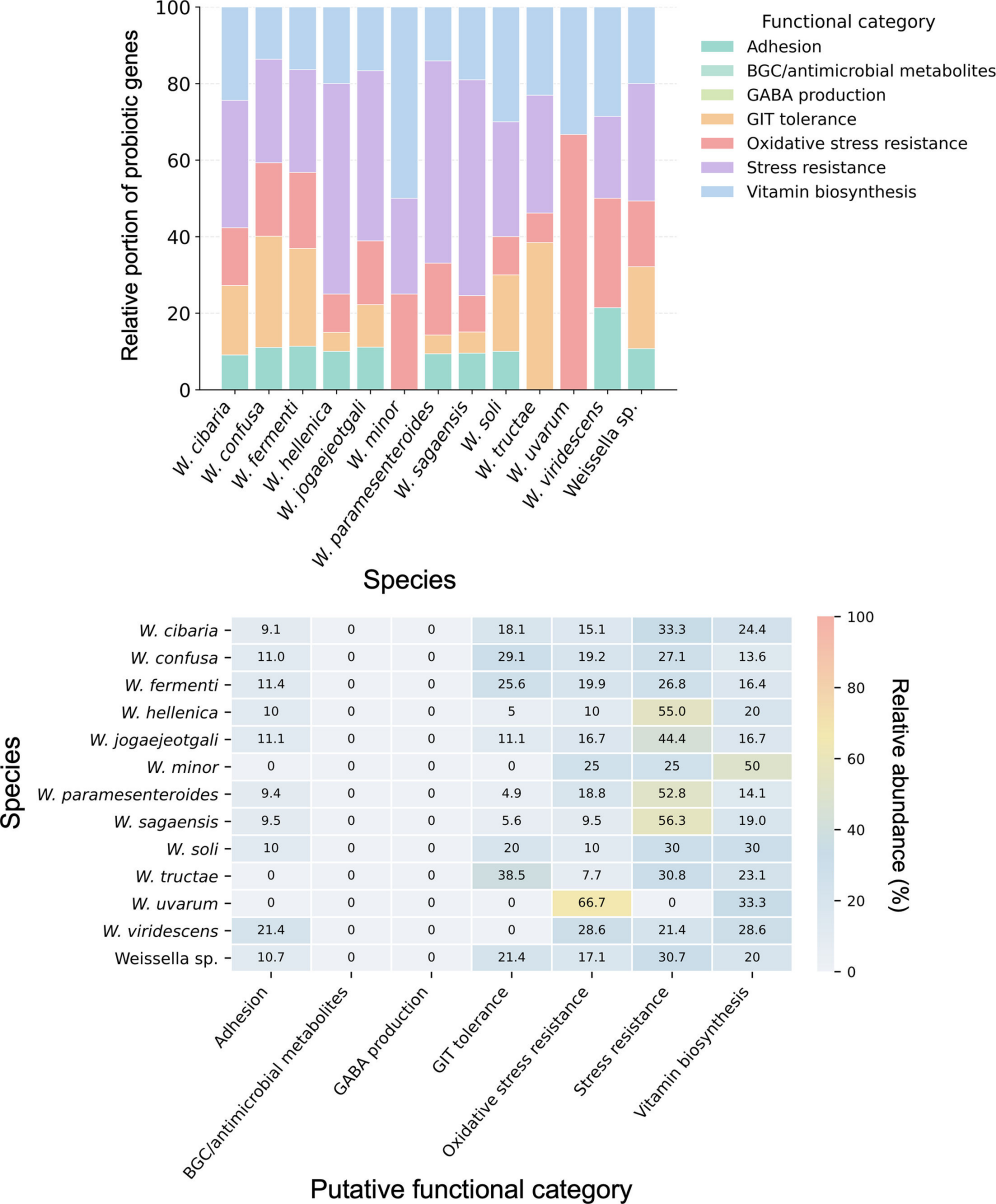
Distribution of putative probiotic-associated genetic potential across *Weissella* species. (**A**) Stacked bar plot showing the relative composition of putative probiotic-associated genetic categories, normalized to 100% within each species. (**B**) Heatmap showing the corresponding percentage contribution of each category across species. Functional categories include adhesion and colonization, BGC and antimicrobial metabolites, GABA production, GIT tolerance, oxidative stress resistance, general stress resistance, and vitamin biosynthesis.

The distribution of putative probiotic-associated genetic categories across *Weissella* species revealed both conserved and species-variable patterns (Fig. 7). After normalization, stress resistance and vitamin biosynthesis-associated genes represented the major components across most species, although their relative contributions varied among taxa. General stress resistance showed particularly high proportions in *W. sagaensis* (56.3%), *W. hellenica* (55.0%), and *W. paramesenteroides* (52.8%), whereas oxidative stress resistance was highest in *W. uvarum* (66.7%). Vitamin biosynthesis-associated genes were also broadly represented, with the highest relative contribution observed in *W. minor* (50.0%), followed by *W. uvarum* (33.3%) and *W. soli* (30.0%).

GIT tolerance-associated genes showed more variable distribution, with higher relative proportions in *W. tructae* (38.5%), *W. confusa* (29.1%), and *W. fermenti* (25.6%). Adhesion-associated genes were present at lower to moderate levels across several species, with relatively higher proportions in *W. viridescens* (21.4%), *W. confusa* (11.0%), and *W. fermenti* (11.4%). In contrast, genes associated with BGC and antimicrobial metabolites and GABA production were not detected in the data set. These findings indicate that putative probiotic-associated genetic potential in *Weissella* is mainly represented by stress response, vitamin biosynthesis, and GIT tolerance markers, whereas adhesion-related traits are more species-variable and specialized metabolite- or GABA-associated markers are absent under the applied screening criteria. These patterns should be interpreted as genomic potential rather than confirmed probiotic phenotype. The putative probiotic markers are listed in Table S4.

### Species-specific putative probiotic-associated genetic profiles of *Weissella*

Species-specific profiles of putative probiotic-associated genetic potential varied across the *Weissella* data set (Fig. S2). Stress resistance, vitamin biosynthesis, GIT tolerance, and oxidative stress resistance represented the major detectable categories, whereas BGC and antimicrobial metabolite-associated genes and GABA production markers were not detected under the applied screening criteria. Among the dominant species, *W. cibaria* and *W. confusa* displayed relatively balanced profiles, combining stress resistance, vitamin biosynthesis, GIT tolerance, oxidative stress resistance, and low-to-moderate adhesion-associated markers (Fig. S2A and B). *W. fermenti* showed a similar pattern, with contributions from GIT tolerance, stress resistance, oxidative stress resistance, vitamin biosynthesis, and adhesion-associated genes (Fig. S2C). In contrast, *W. hellenica, W. jogaejeotgali, W. paramesenteroides*, and *W. sagaensis* were more strongly weighted toward general stress resistance, suggesting greater representation of stress-associated genetic potential (Fig. S2D, E, G and H). Several species showed more restricted or category-skewed profiles. *W. minor* was mainly represented by vitamin biosynthesis, stress resistance, and oxidative stress resistance markers, whereas *W. tructae* showed a higher relative contribution of GIT tolerance-associated genes (Fig. S2F and J). *W. uvarum* was dominated by oxidative stress resistance and vitamin biosynthesis markers, while *W. viridescens* showed a comparatively higher contribution of adhesion and oxidative stress resistance markers (Fig. S2K and L). The unclassified *Weissella* sp. group displayed a mixed profile including GIT tolerance, stress resistance, oxidative stress resistance, vitamin biosynthesis, and adhesion-associated markers (Fig. S2M). These patterns indicate that putative probiotic-associated genetic potential is unevenly distributed across *Weissella* species and should be interpreted as genome-predicted potential rather than confirmed probiotic phenotype.

### The EPS biosynthesis cluster among *Weissella*

**Fig 8:**
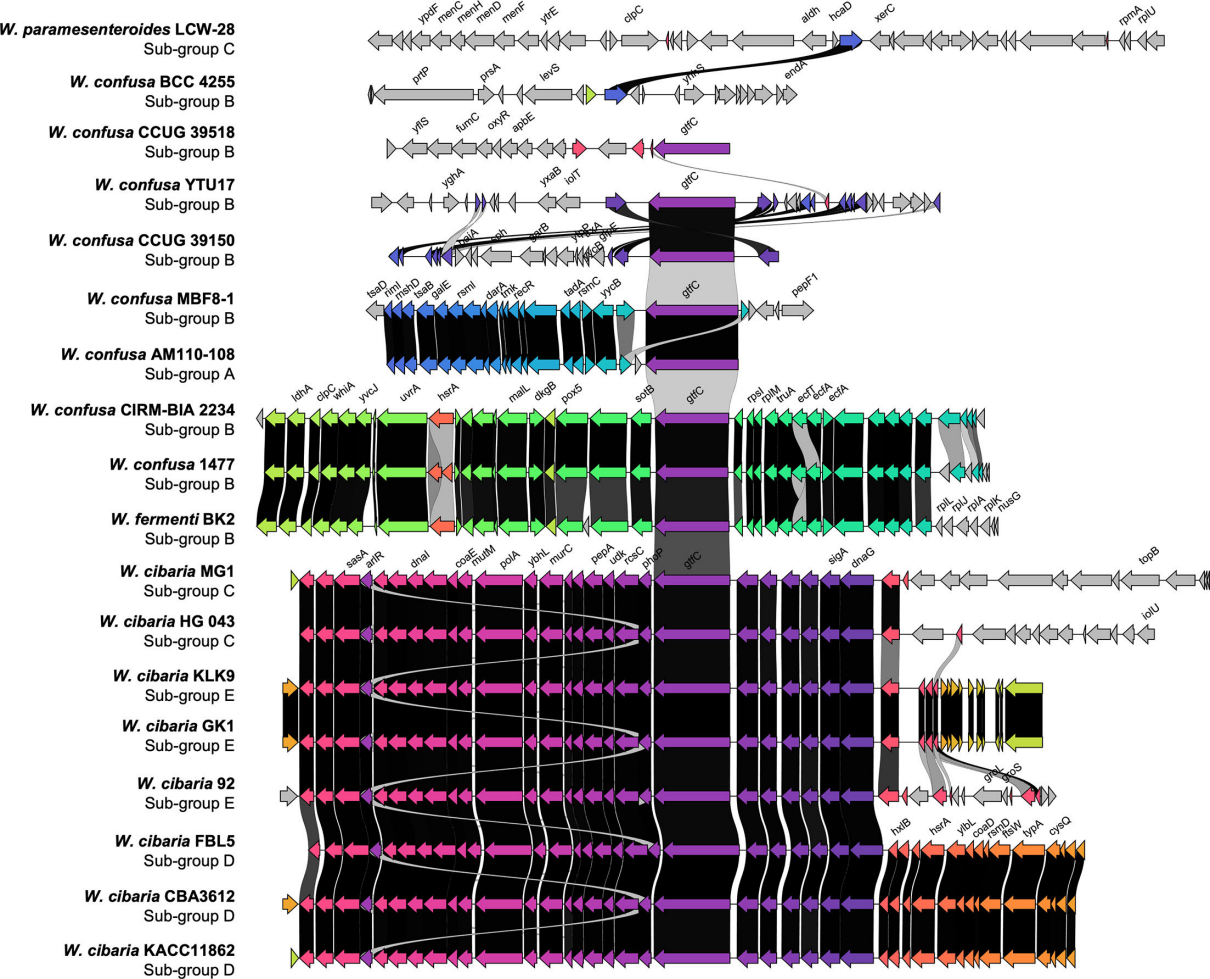
Synteny analysis of representative EPS loci in *Weissella* species. The synteny plot shows six sub-groups of EPS loci based on gene order and functional content. Sub-group A features loci with IS elements, and sub-group B contains conserved loci with few sugar-modifying enzymes. Sub-group C has enriched loci with regulators and sugar-modifying enzymes. Sub-group D shows full-length loci with glycosyltransferases and regulators. Sub-group E is marked by high IS elements and rearrangements, whereas sub-group F consists of compact loci with a high proportion of hypothetical proteins and fewer EPS-related enzymes.

The synteny analysis exhibits the organization of EPS biosynthesis loci across various *Weissella* strains, grouped into six sub-groups of 286 clusters based on their gene order patterns. The representative clusters of each sub-group are presented in Fig. 8. Sub-group A contains medium-sized loci with insertion sequences (IS), found in strains such as *W. confusa*. Sub-group B includes full-length loci with conserved gene order, but fewer predicted EPS-associated enzymatic components, predominantly in *W. confusa* and some *W. fermenti*. Sub-group C contains full-length loci more frequently observed with sugar-modifying enzymes and regulators, suggesting genomic potential for EPS biosynthesis, mainly found in *W. cibaria*. Sub-group D represents full-length loci with a high presence of glycosyltransferases and regulators, indicating putatively complete EPS biosynthesis loci, primarily in *W. cibaria*. Sub-group E includes full-length loci with a high presence of insertion sequences and transposases, suggesting ongoing recombination and structural rearrangements. However, sub-group F consists of compact loci with a high proportion of hypothetical proteins and fewer EPS-related enzymes. The summary of each synteny category is presented in Table S3. Statistical analysis revealed significant species-associated enrichment of specific EPS synteny sub-groups. Sub-group D was strongly over-represented in *W. cibaria* (*q* = 2.72 × 10^−44^), while sub-group B was enriched in *W. confusa* (*q* = 2.17 × 10^−35^). Additional significant associations included sub-group C in *W. cibaria* (*q* = 2.65 × 10^−4^) and sub-group F in *W. confusa* (*q* = 2.65 × 10^−4^). These results indicate non-random, species-associated distribution patterns of EPS gene cluster architectures.

### Prevalence of biosynthesis gene cluster in *Weissella*

**Fig 9:**
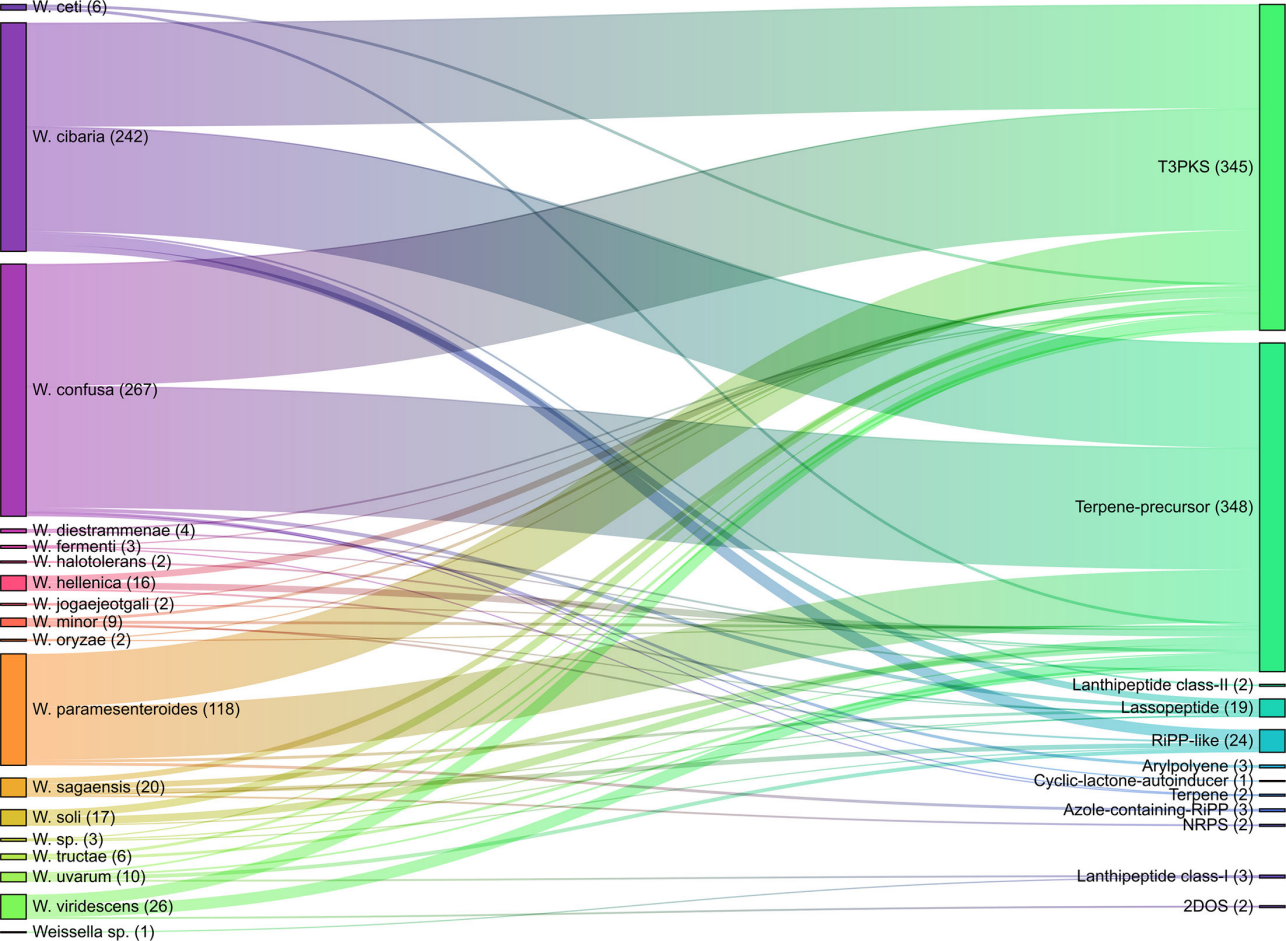
Distribution of secondary metabolite BGCs across *Weissella* species. The Sankey diagram illustrates the presence of secondary metabolite BGCs in *Weissella* species. Species are represented on the left, and metabolites on the right, with links showing the presence of gene clusters. Thicker links indicate higher abundance, highlighting core metabolites like T3PKS and terpene-precursor.

The prevalence of secondary metabolite BGCs was analyzed through antiSMASH v.8 across various *Weissella* species. The most common secondary metabolites identified across the species were T3PKS (polyketide synthases) and terpene-precursor clusters, both of which appeared in the majority of species, including *W. cibaria* and *W. confusa*. Other secondary metabolites, such as RiPP-like, lassopeptides, and lanthipeptides, were found in specific species, such as *W. cibaria* and *W. paramesenteroides*. While these metabolites were less widespread compared to T3PKS and terpene precursors, they were still prevalent within certain species. Interestingly, some species, including *W. viridescens*, *W. confusa*, and *W. paramesenteroides* exhibited a more restricted repertoire of secondary metabolites. These species exhibited fewer gene clusters, which were often species-specific, such as 2DOS, arylpolyene, and azole-containing RiPPs. These rare metabolites were mostly found in only a few *Weissella* species (Fig. 9). However, no bacteriocin-associated gene clusters were identified by BAGEL4 in the analyzed Weissella genomes, and thus, the BGC results presented here are limited to antiSMASH-predicted secondary metabolite categories.

## DISCUSSION

**Fig 10:**
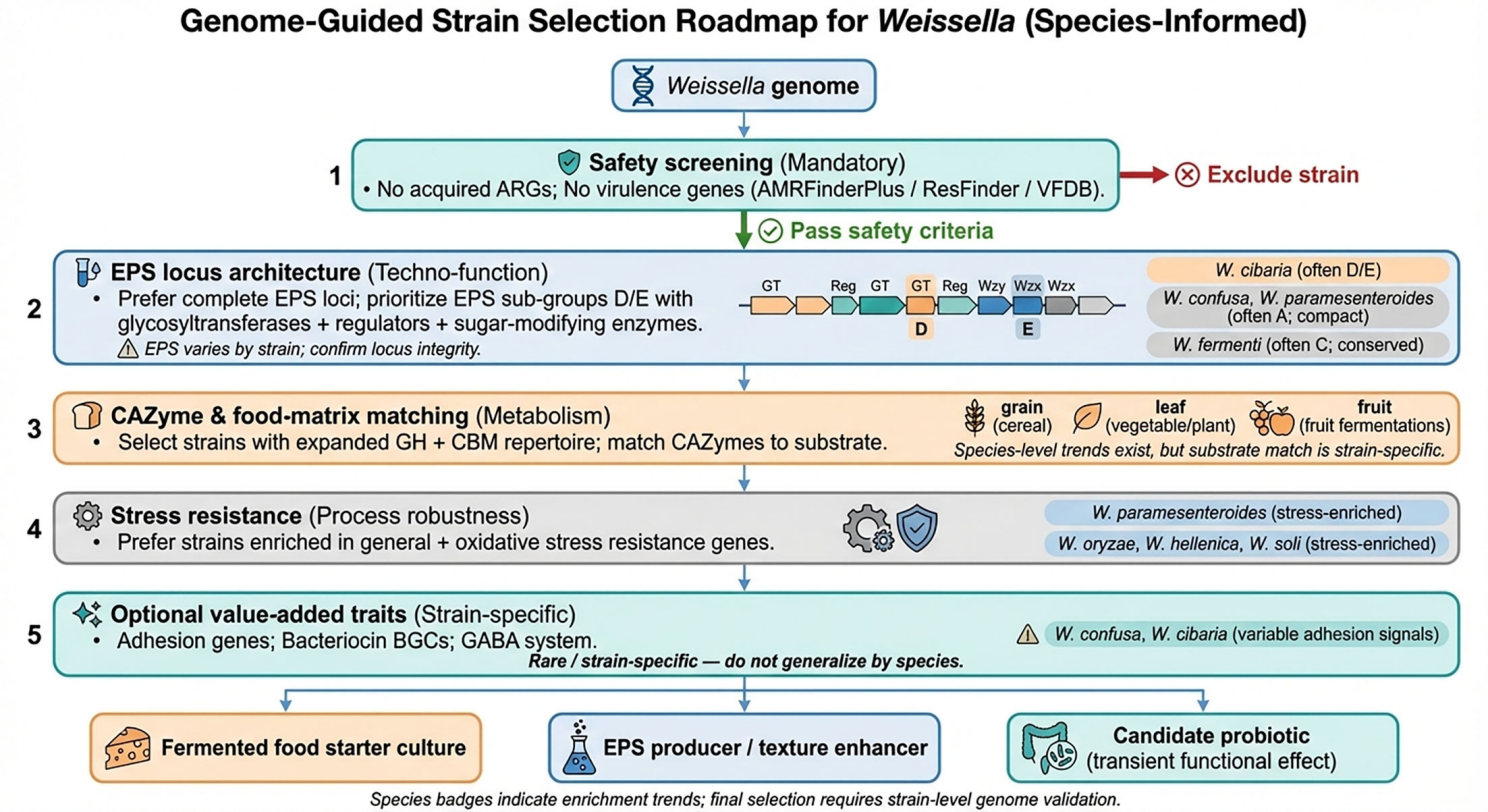
Conceptual genome-guided framework for preliminary *Weissella* strain prioritization. The framework summarizes *in silico* criteria for guiding candidate selection, including ARG-virulence determinant screening, EPS biosynthesis potential, carbohydrate metabolism, stress resistance, and optional value-added traits. Species labels indicate data set-level prevalence trends rather than validated species properties. Final strain selection requires strain-level genomic review and phenotypic confirmation.

The genus *Weissella* comprises heterofermentative LABs widely distributed across fermented foods, plant-associated environments, and animal- and human-related niches (28). Comparative genomic analysis confirmed substantial interspecies diversity in genome size, GC content, and accessory gene content, highlighting pronounced evolutionary divergence within the genus *Weissella* (29). The clear species-specific clustering in genome size-GC content space suggests that these genomic traits are relatively stable within species and reflect long-term lineage differentiation rather than recent homogenization (30, 31). Smaller genomes, such as in *W. tructae*, may indicate niche specialization, whereas larger genomes, such as in *W. cibaria*, may support greater metabolic flexibility (32, 33). Similar patterns have been reported in other LABs, where genome expansion is linked to broader ecological adaptability and carbohydrate utilization capacity (34). Although *W. confusa* and *W. cibaria* dominated the public data set, likely reflecting both ecological prevalence and sampling intensity, the consistent species-level clustering supports the quality of genome curation and the reliability of this data set for downstream comparative analyses. For the species identification, the AAI-based clustering revealed sharp species boundaries across the *Weissella* genus, with high within-species similarity and clear separation between species-level groups. The presence of well-defined diagonal blocks corresponding to major species, including *W. confusa*, *W. cibaria*, and *W. paramesenteroides,* confirms the genomic coherence of these taxa. Smaller but distinct clusters representing less abundant species further demonstrate that AAI remains a sensitive and reliable metric for delineating species boundaries even in unevenly represented data sets (35). Interestingly, the grouping of certain species pairs, such as *W. confusa* and *W. fermenti* and *W. hellenica* with *W. sagaensis*, suggests closer evolutionary relationships that may warrant further taxonomic or functional investigation. These relationships are consistent with their proximity in genome architecture and accessory gene content and highlight the value of whole-genome metrics over single-gene phylogenies for resolving fine-scale taxonomic structure within *Weissella* (36). To translate comparative genomic insights into practical *Weissella* strain selection, a genome-guided, species-informed decision framework was developed (Fig. 10). This framework integrates safety, metabolic capacity, and techno-functional potential into a sequential selection process while maintaining a clear emphasis on strain-level validation. The first and most essential selection step is genomic safety screening, in which strains are evaluated for the absence of acquired ARGs and virulence factor-associated determinants, both of which are critical considerations for food, industrial, and probiotic applications (37, 38). In the present data set, ARGs were detected in only three strains, and no virulence factor-associated genes were identified, indicating that such risk determinants are rare and strain-specific rather than genus-wide. This highlights the importance of genome-based screening at the strain level rather than assuming safety based solely on genus assignment (39–41). Following safety clearance, EPS biosynthesis potential represents a key techno-functional decision point. Comparative synteny analysis revealed multiple EPS locus architectures across the genus, allowing species-informed prioritization. Strains of *W. cibaria* are frequently associated with complete EPS loci, higher proportions of glycosyltransferases, regulatory elements, and sugar-modifying enzymes, indicating a greater genomic potential for structurally diverse EPS biosynthesis (42). In contrast, *W. confusa* and *W. paramesenteroides* more often carried compact or conserved loci, indicating more limited or variable EPS potential. These patterns suggest that EPS operons in *Weissella* are evolutionarily dynamic, likely shaped in part by horizontal gene transfer and mobile genetic elements (43) and may therefore serve as useful preliminary markers when selecting strains for texture- or viscosity-related applications.

The next decision layer evaluates carbohydrate metabolic capacity through the abundance and diversity of CAZyme-associated genes, which provide insight into metabolic potential and ecological adaptation in *Weissella* (44). Across the genus, CAZyme repertoires were dominated by GH and GT families, indicating a conserved carbohydrate-processing backbone. The widespread presence of GH13-related enzymes together with GH32 and GH70 suggests a shared capacity to utilize starch, sucrose, and glucans commonly found in fermented food environments (45). Broad conservation of GT families further supports essential roles in cell wall biogenesis and EPS synthesis. Species-level variation was mainly observed in accessory CAZymes. *W. cibaria* showed relatively higher GT representation, consistent with its reported roles in EPS production and food texture development (46), whereas *W. confusa* carried more CBMs, suggesting broader carbohydrate-binding capacity and ecological versatility (47). In contrast, AAs and PLs were limited across the genus, supporting a predominantly fermentative lifestyle rather than oxidative polysaccharide degradation (48). These findings indicate that strain selection should consider compatibility between CAZyme profiles and the intended food matrix rather than relying solely on species identity. The distribution of putative probiotic-associated genetic markers in *Weissella* indicates that stress response, vitamin biosynthesis, oxidative stress resistance, and GIT tolerance represent the major detectable functional categories in the *Weissella* data set. General stress resistance markers were proportionally high in *W. sagaensis*, *W. hellenica*, and *W. paramesenteroides*, suggesting that these taxa may possess greater genomic capacity for coping with processing-related stresses such as acidity, osmotic pressure, and oxidative exposure (49, 50). Oxidative stress resistance was particularly prominent in *W. uvarum*, whereas GIT tolerance-associated markers were more represented in *W. tructae*, *W. confusa*, and *W. fermenti*. Vitamin biosynthesis-associated genes were broadly detected, suggesting potential contribution to micronutrient availability in fermented foods (51). In contrast, adhesion-associated markers were generally limited and species-variable, with relatively higher proportions in *W. viridescens*, *W. fermenti*, and *W. confusa*, indicating that host-interaction potential is not a conserved genus-wide feature (52–54). However, BGC and antimicrobial metabolite-associated markers and GABA production genes were not detected in the data set, suggesting that these functions are absent or highly restricted under the applied criteria. However, these profiles represent putative genetic potential inferred from gene presence and do not confirm probiotic activity. Gene expression, regulation, environmental conditions, and strain-specific physiology may influence whether these traits are functional. Therefore, these results should be used for preliminary genome-guided strain prioritization, with phenotypic validation required to confirm stress tolerance, adhesion, metabolite production, safety, and probiotic performance. The biosynthetic diversity of *Weissella* species highlights both shared and species-specific pathways for secondary metabolite production. The widespread occurrence of T3PKS and terpene-precursor clusters across many species suggests that these pathways may contribute to ecological fitness, microbial competition, and environmental resilience in niches such as fermented foods and animal-associated habitats (55, 56). In contrast, RiPP-like clusters, lassopeptides, and lanthipeptides were detected in fewer taxa, particularly *W. cibaria* and *W. paramesenteroides*, indicating potential antimicrobial peptide production in selected lineages (57). More specialized clusters, including 2DOS, arylpolyene, and azole-containing RiPPs, were rare and restricted to a small number of genomes, suggesting niche-specific ecological functions such as defense or signaling (58). These findings indicate that *Weissella* possesses broader biosynthetic potential than expected for a typical fermentative genus. However, because these traits were unevenly distributed across species and strains, they should be considered strain-specific opportunities rather than universal species characteristics. As with other genome-predicted probiotic traits, experimental validation remains necessary to confirm functionality and application value.

In addition, Heap’s law analysis supported an open pan-genome in *Weissella*, with a positive exponent indicating continued gene accumulation as more genomes are sampled (59). This is consistent with the overall pan-genome structure, which was dominated by shell and cloud genes and contained only a small conserved core, highlighting substantial accessory genome diversity. Such a pattern suggests marked genomic plasticity and indicates that adaptation in *Weissella* is driven largely by lineage- and strain-specific gene repertoires (60). Open pan-genomes are typical of bacteria occupying heterogeneous environments, where variable ecological pressures promote gene gain and functional diversification (61). The core genome phylogeny showed clear species-level clustering across *Weissella*, supporting the reliability of genome-based classification and confirming that conserved genes retain sufficient signal to resolve major lineages (62). When the same tree was integrated with the pan-genome presence-absence matrix, closely related lineages also tended to share similar gene-content patterns, indicating concordance between core genome ancestry and accessory genome composition. Large clades such as *W. confusa* and *W. cibaria* displayed broad blocks of shared gene clusters together with variable accessory regions, suggesting conserved species backbones accompanied by substantial strain-level diversification. In contrast, smaller or less represented species formed narrower and more distinct matrix patterns, consistent with reduced sampling depth or more specialized gene repertoires. Closely related species pairs, such as *W. confusa* with *W. fermenti* and *W. hellenica* with *W. sagaensis*, showed partially similar pan-genome signatures, supporting their close evolutionary relationship. Notably, genomes annotated as *Weissella* sp. formed a distinct lineage with unique gene-content patterns, suggesting unresolved taxonomic diversity and potential novel species candidates. The combined phylogenomic and pan-genomic patterns indicate that *Weissella* is a genomically coherent but highly flexible genus, where species-level structure is maintained despite extensive accessory genome variation. Notably, genomes designated as *Weissella* sp. did not cluster within any established species clade but instead formed a separate lineage distinct from recognized taxa. This phylogenomic placement suggests that these unclassified genomes may represent misidentified strains, taxonomically unresolved members, or potentially a novel *Weissella* species pending further characterization. Metadata integration showed that isolation source and geography were not strictly lineage-specific. Fermented food isolates were widely distributed, especially within *W. confusa* and *W. cibaria*, whereas animal-, human-, insect-, environmental-, and raw food-associated isolates were scattered across multiple clades. *W. confusa* displayed the broadest ecological range, supporting a generalist profile, while *W. cibaria* was mainly associated with fermented foods. Major species also showed broad geographic distribution, whereas rare taxa were often limited to one or a few countries, likely reflecting sampling bias rather than true geographic restriction (63, 64). These findings indicate that the open pan-genome of *Weissella* reflects ongoing gene acquisition driven by ecological diversity, niche adaptation, and strain-level specialization across environments and regions. Therefore, these findings highlight the genomic flexibility and ecological adaptability of *Weissella*, with significant implications for its application in fermentation, probiotics, and biotechnology, while emphasizing the importance of strain-level validation for accurate functional predictions and optimal strain selection.

### Conclusion

This study provides a comprehensive genomic framework for understanding the diversity and functional potential of *Weissella* species, offering new insights into their ecological adaptation and capacity to produce bioactive compounds relevant to food fermentation, probiotic development, and biotechnology. The strain-level analyses emphasize the importance of genome-guided strain selection, particularly in relation to safety assessment and key functional traits such as exopolysaccharide biosynthesis, carbohydrate metabolism, and stress tolerance, which are critical for performance and stability in industrial applications. The identification of an open pan-genome further highlights the evolutionary plasticity of the genus and its potential as a reservoir for novel functional genes and bioactive metabolites. Therefore, these findings advance our understanding of *Weissella* at both the species and strain levels and support a rational, evidence-based approach to strain selection. This work lays a solid foundation for future investigations into the ecological roles of *Weissella* across diverse environments and for the targeted exploitation of selected strains in functional foods, health-related applications, and sustainable biotechnological innovations.

## Data Availability

All genome assemblies analyzed in this study are publicly available from NCBI RefSeq, and accession numbers with associated metadata are provided in Table S1. No new genome sequence data were generated in this study.

## SUPPLEMENTAL MATERIAL

Supplemental material spectrum.00331-26-s0001.docx Fig. S1 and S2; Tables S1 to S4.

ASM does not own the copyrights to Supplemental Material that may be linked to, or accessed through, an article. The authors have granted ASM a non-exclusive, world-wide license to publish the Supplemental Material files. Please contact the corresponding author directly for reuse.
