# Supplementary material for: Genome-guided characterization of Weissella species highlights strain-specific functional traits relevant to food fermentation and probiotics
Source: Microbiol Spectr. 2026 Jul 13;14(8):e00331-26. doi: 10.1128/spectrum.00331-26 (PMC13436363; doi:10.1128/spectrum.00331-26)
Supplement: Supplementary file 1 [file spectrum.00331-26-s0001.docx]

**Figure S1. Pan-genome analysis of 347 Weissella genomes using Panaroo.**
**(A)** Pan-genome accumulation curve showing an open pan-genome structure, fitted by Heap’s law model. **(B)** Gene frequency distribution across genomes, illustrating that many gene clusters are present in only a subset of genomes. **(C)** Distribution of core, soft-core, shell, and cloud genes, comprising **23,496** total gene clusters, including **29 core genes, 40 soft-core genes, 4,025 shell genes,** and **19,402 cloud genes.**

**Figure S2.** **Species-specific putative probiotic-associated genetic profiles of *Weissella*.** Radar plots show the relative distribution of putative probiotic-associated genetic categories across species. Each plot represents one species based on the updated Panaroo dataset: (A) *W. cibaria*, (B) *W. confusa*, (C) *W. fermenti*, (D) *W. hellenica*, (E) *W. jogaejeotgali*, (F) *W. minor*, (G*) W. paramesenteroides*, (H) *W. sagaensis*, (I) *W. soli*, (J) *W. tructae*, (K) *W. uvarum*, (L) *W. viridescens*, and (M) *Weissella* sp. Values indicate genomic potential only.

**Table S1** Metadata of available *Weissella* genomes in the NCBI database used in this study.

| **Accession number** | **Species** | | **Strain** | | | **Isolation source category** | | **Country** | | **Sequence length (bp)** | | **Number of contigs** | **GC (%)** |
| --- | --- | --- | --- | --- | --- | --- | --- | --- | --- | --- | --- | --- | --- |
| GCF_000193635.1 | *W. cibaria* | | KACC 11862 | | | Fermented Food | | Korea | | 1,352,640 | | 7 | 41 |
| GCF_000878185.1 | *W. cibaria* | | AB3b | | | Fermented Food | | Ireland | | 1,358,385 | | 8 | 43 |
| GCF_000878205.1 | *W. cibaria* | | MG1 | | | Fermented Food | | Ireland | | 2,129,476 | | 72 | 39 |
| GCF_000878215.1 | *W. cibaria* | | ff3PR | | | Animal | | Ireland | | 1,355,853 | | 1 | 41 |
| GCF_001308145.2 | *W. cibaria* | | CH2 | | | Fermented Food | | India | | 1,389,513 | | 1 | 40.5 |
| GCF_001614295.1 | *W. cibaria* | | FBL5 | | | Fermented Food | | South Korea | | 1,390,397 | | 1 | 40.5 |
| GCF_001932695.1 | *W. cibaria* | | CMS2 | | | Human | | South Korea | | 2,465,158 | | 108 | 44.5 |
| GCF_001951075.2 | *W. cibaria* | | CMU | | | Human | | South Korea | | 2,436,232 | | 58 | 44.5 |
| GCF_001951095.1 | *W. cibaria* | | CMS3 | | | Human | | South Korea | | 2,357,128 | | 60 | 45 |
| GCF_002115575.1 | *W. cibaria* | | DmW_103 | | | Animal | | USA | | 2,577,731 | | 7 | 44.5 |
| GCF_002243305.1 | *W. cibaria* | | CMS1 | | | Human | | South Korea | | 1,366,073 | | 27 | 43 |
| GCF_002614625.1 | *W. cibaria* | | 110 | | | Fermented Food | | Thailand | | 2,218,304 | | 82 | 44.5 |
| GCF_003010455.1 | *W. cibaria* | | BM2 | | | Fermented Food | | Korea | | 1,537,173 | | 34 | 41 |
| GCF_003076495.1 | *W. cibaria* | | M2 | | | Animal | | China | | 1,768,499 | | 50 | 39.5 |
| GCF_003469955.1 | *W. cibaria* | | AM27-24 | | | Human | | China | | 2,319,355 | | 39 | 45 |
| GCF_003469985.1 | *W. cibaria* | | AM27-22LB | | | Human | | China | | 1,683,184 | | 1 | 43.5 |
| GCF_004103655.1 | *W. cibaria* | | SRCM103448 | | | Food | | South Korea | | 2,181,636 | | 44 | 44.5 |
| GCF_004521955.1 | *W. cibaria* | | SP19 | | | Animal | | Kuwait | | 2,149,545 | | 2 | 39 |
| GCF_005405525.1 | *W. cibaria* | | JCM 12495 | | | Food | | Malaysia | | 2,381,468 | | 2 | 45.5 |
| GCF_006517775.1 | *W. cibaria* | | CBA3612 | | | Fermented Food | | South Korea | | 2,363,492 | | 2 | 45.5 |
| GCF_007785585.1 | *W. cibaria* | | BC10 | | | Fermented Food | | South Korea | | 2,465,612 | | 34 | 45 |
| GCF_007785635.1 | *W. cibaria* | | GGM1 | | | Fermented Food | | South Korea | | 2,342,849 | | 1 | 45.5 |
| GCF_007785645.1 | *W. cibaria* | | BC14 | | | Fermented Food | | South Korea | | 1,947,910 | | 1 | 38 |
| GCF_007785675.1 | *W. cibaria* | | GK3 | | | Fermented Food | | South Korea | | 2,347,049 | | 36 | 45 |
| GCF_007785685.1 | *W. cibaria* | | GK1 | | | Fermented Food | | South Korea | | 2,473,510 | | 2 | 45 |
| GCF_007785715.1 | *W. cibaria* | | DK12 | | | Fermented Food | | South Korea | | 2,610,714 | | 3 | 45 |
| GCF_007785725.1 | *W. cibaria* | | GGI4 | | | Fermented Food | | South Korea | | 2,284,353 | | 52 | 44.5 |
| GCF_007785755.1 | *W. cibaria* | | GGM13-2 | | | Fermented Food | | South Korea | | 1,658,396 | | 8 | 43.5 |
| GCF_007785875.1 | *W. cibaria* | | KLK9 | | | Fermented Food | | South Korea | | 2,346,332 | | 38 | 44.5 |
| GCF_008691985.1 | *W. cibaria* | | cf-23 | | | Food | | Bangladesh | | 2,505,652 | | 21 | 45 |
| GCF_008691995.1 | *W. cibaria* | | CT-37 | | | Food | | Bangladesh | | 2,506,353 | | 21 | 45 |
| GCF_009864045.1 | *W. cibaria* | | 10M | | | Fermented Food | | Italy | | 1,612,814 | | 19 | 41.5 |
| GCF_011009635.1 | *W. cibaria* | | A23 | | | Animal | | United Kingdom | | 2,453,054 | | 4 | 45 |
| GCF_012277025.1 | *W. cibaria* | | 92 | | | Fermented Food | | India | | 2,039,164 | | 11 | 38.5 |
| GCF_012277235.1 | *W. cibaria* | | AV1 | | | Fermented Food | | India | | 2,504,603 | | 55 | 45 |
| GCF_014061105.1 | *W. cibaria* | | YRK005 | | | Food | | South Korea | | 2,010,213 | | 101 | 44.5 |
| GCF_014081885.1 | *W. cibaria* | | CBA3636 | | | Unknown | | South Korea | | 2,270,650 | | 46 | 44.5 |
| GCF_014694565.1 | *W. cibaria* | | UC4051 | | | Fermented Food | | Italy | | 2,276,169 | | 40 | 44.5 |
| GCF_015551705.1 | *W. cibaria* | | 1001713B170221_170320_B3 | | | Human | | USA | | 2,196,755 | | 25 | 44.5 |
| GCF_015552515.1 | *W. cibaria* | | 1001283B150209_150212_A5 | | | Human | | USA | | 2,233,286 | | 37 | 44.5 |
| GCF_015558475.1 | *W. cibaria* | | D6t1_180914_E9 | | | Human | | USA | | 2,312,481 | | 34 | 45 |
| GCF_015559665.1 | *W. cibaria* | | 1001287H_170206_A9 | | | Human | | USA | | 2,221,895 | | 60 | 44.5 |
| GCF_018991795.1 | *W. cibaria* | | 7.8.34 | | | Fermented Food | | USA | | 2,322,300 | | 39 | 44.5 |
| GCF_018993825.1 | *W. cibaria* | | 3.8.44 | | | Fermented Food | | USA | | 2,272,732 | | 75 | 44.5 |
| GCF_020089765.1 | *W. cibaria* | | KCKM 0342 | | | Fermented Food | | South Korea | | 2,318,994 | | 88 | 44.5 |
| GCF_020183895.1 | *W. cibaria* | | D-H/1MSE W25 | | | Environment | | Brazil | | 2,179,627 | | 17 | 45 |
| GCF_020540475.1 | *W. cibaria* | | MSK.22.139 | | | Human | | USA | | 2,384,251 | | 41 | 44.5 |
| GCF_020540505.1 | *W. cibaria* | | MSK.22.138 | | | Human | | USA | | 2,215,045 | | 95 | 45 |
| GCF_020540525.1 | *W. cibaria* | | MSK.22.130 | | | Human | | USA | | 2,255,217 | | 40 | 44.5 |
| GCF_020540565.1 | *W. cibaria* | | MSK.22.122 | | | Human | | USA | | 2,267,867 | | 33 | 44.5 |
| GCF_020540585.1 | *W. cibaria* | | MSK.22.127 | | | Human | | USA | | 2,246,744 | | 139 | 45 |
| GCF_020540595.1 | *W. cibaria* | | MSK.22.123 | | | Human | | USA | | 2,178,881 | | 13 | 45 |
| GCF_020540625.1 | *W. cibaria* | | MSK.22.119 | | | Human | | USA | | 2,340,566 | | 127 | 45 |
| GCF_020540635.1 | *W. cibaria* | | MSK.22.120 | | | Human | | USA | | 2,215,163 | | 46 | 44.5 |
| GCF_020553615.1 | *W. cibaria* | | MSK.22.134 | | | Human | | USA | | 2,216,758 | | 2 | 45 |
| GCF_020709095.1 | *W. cibaria* | | MSK.22.132 | | | Human | | USA | | 2,373,290 | | 4 | 45 |
| GCF_020844635.1 | *W. cibaria* | | FUA3585 | | | Fermented Food | | Zimbabwe | | 1,934,584 | | 34 | 36.5 |
| GCF_021029605.1 | *W. cibaria* | | LMG 21843 | | | Fermented Food | | South Korea | | 2,323,953 | | 25 | 45 |
| GCF_021513015.1 | *W. cibaria* | | CXO-1 | | | Human | | South Korea | | 1,814,617 | | 32 | 37 |
| GCF_022751235.1 | *W. cibaria* | | JW15 | | | Fermented Food | | South Korea | | 1,977,810 | | 22 | 38 |
| GCF_023008225.1 | *W. cibaria* | | NH9449 | | | Fermented Food | | Thailand | | 2,457,678 | | 4 | 45 |
| GCF_024586845.1 | *W. cibaria* | | NM1 | | | Animal | | Malaysia | | 1,480,318 | | 22 | 41 |
| GCF_024723295.1 | *W. cibaria* | | HG043 | | | Human | | India | | 1,808,244 | | 45 | 37 |
| GCF_025121995.1 | *W. cibaria* | | LBAE H25 | | | Fermented Food | | France | | 2,510,480 | | 5 | 45 |
| GCF_025122015.1 | *W. cibaria* | | LBAE K39 | | | Fermented Food | | France | | 2,427,225 | | 2 | 45 |
| GCF_025122045.1 | *W. cibaria* | | LBAE D39 | | | Fermented Food | | France | | 2,513,229 | | 3 | 45 |
| GCF_025127215.1 | *W. cibaria* | | 1258 | | | Fermented Food | | Cote d'Ivoire | | 2,439,082 | | 3 | 45 |
| GCF_025128955.1 | *W. cibaria* | | 1364 | | | Fermented Food | | Cote d'Ivoire | | 2,543,107 | | 3 | 45 |
| GCF_025129035.1 | *W. cibaria* | | 1522 | | | Fermented Food | | Mali | | 2,557,969 | | 4 | 45 |
| GCF_025129155.1 | *W. cibaria* | | 1268 | | | Fermented Food | | Cote d'Ivoire | | 2,571,555 | | 2 | 45 |
| GCF_025132715.1 | *W. cibaria* | | 1374 | | | Fermented Food | | Cote d'Ivoire | | 2,494,749 | | 3 | 45 |
| GCF_025132755.1 | *W. cibaria* | | 1369 | | | Fermented Food | | Cote d'Ivoire | | 2,561,573 | | 3 | 45 |
| GCF_025132795.1 | *W. cibaria* | | 1365 | | | Fermented Food | | Cote d'Ivoire | | 1,731,541 | | 5 | 43.5 |
| GCF_025395315.1 | *W. cibaria* | | 758 | | | Food | | Australia | | 1,655,187 | | 12 | 43.5 |
| GCF_025395435.1 | *W. cibaria* | | 752 | | | Food | | Australia | | 2,192,057 | | 41 | 44.5 |
| GCF_025617335.1 | *W. cibaria* | | 22 | | | Food | | Australia | | 2,000,982 | | 25 | 38 |
| GCF_025770435.1 | *W. cibaria* | | 7.8.34 | | | Fermented Food | | USA | | 2,001,409 | | 24 | 38 |
| GCF_027667085.1 | *W. cibaria* | | AM66-48ACA | | | Human | | China | | 2,001,555 | | 28 | 38 |
| GCF_027672495.1 | *W. cibaria* | | AM113-88 | | | Human | | China | | 2,001,563 | | 27 | 38 |
| GCF_027672605.1 | *W. cibaria* | | AM113-89 | | | Human | | China | | 2,002,052 | | 24 | 38 |
| GCF_027678285.1 | *W. cibaria* | | TM115-250 | | | Human | | China | | 1,931,304 | | 21 | 38 |
| GCF_027723405.1 | *W. cibaria* | | AM110-02 | | | Human | | China | | 1,993,100 | | 49 | 38 |
| GCF_028201435.1 | *W. cibaria* | | BAL3C-5 C120T | | | Fermented Food | | Spain | | 1,966,926 | | 26 | 38 |
| GCF_028201455.1 | *W. cibaria* | | BAL3C-5 | | | Fermented Food | | Spain | | 1,990,007 | | 47 | 38 |
| GCF_029872315.1 | *W. cibaria* | | R9-1 | | | Fermented Food | | China | | 1,992,930 | | 48 | 38 |
| GCF_030247095.1 | *W. cibaria* | | ATP210 | | | Animal | | Thailand | | 1,992,671 | | 50 | 38 |
| GCF_030851325.1 | *W. cibaria* | | A-H/7 W21 | | | Environment | | Brazil | | 1,964,202 | | 48 | 38 |
| GCF_030851365.1 | *W. cibaria* | | D-S/4 W42 | | | Environment | | Brazil | | 1,990,597 | | 47 | 38 |
| GCF_033174705.1 | *W. cibaria* | | com2 | | | Fermented Food | | Italy | | 2,018,582 | | 23 | 38 |
| GCF_034083555.1 | *W. cibaria* | | YTU5 | | | Fermented Food | | Turkey | | 1,991,534 | | 48 | 38 |
| GCF_039059255.1 | *W. cibaria* | | 2227st1_B11_2227SCRN_220401 | | | Human | | USA | | 2,353,948 | | 48 | 45 |
| GCF_040267675.1 | *W. cibaria* | | CXO-1 | | | Human | | South Korea | | 2,376,380 | | 43 | 45 |
| GCF_041147085.1 | *W. cibaria* | | 7.8.48 | | | Fermented Food | | USA | | 2,426,251 | | 16 | 45 |
| GCF_041147175.1 | *W. cibaria* | | 7.8.34 | | | Fermented Food | | USA | | 2,239,284 | | 151 | 45 |
| GCF_041167665.1 | *W. cibaria* | | KM26 | | | Fermented Food | | South Korea | | 1,867,525 | | 20 | 36.5 |
| GCF_041927265.1 | *W. cibaria* | | ZJU.1 | | | Fermented Food | | China | | 1,920,171 | | 28 | 36.5 |
| GCF_042472175.1 | *W. cibaria* | | HY207 | | | Fermented Food | | South Korea | | 1,943,601 | | 20 | 38 |
| GCF_042692105.1 | *W. cibaria* | | BWL4 | | | Food | | China | | 1,940,816 | | 20 | 38 |
| GCF_044068405.1 | *W. cibaria* | | 1BM | | | Animal | | Brazil | | 2,455,642 | | 62 | 45 |
| GCF_046592615.1 | *W. cibaria* | | TATVAM-FAB27 | | | Animal | | India | | 2,332,391 | | 4 | 45 |
| GCF_046592715.1 | *W. cibaria* | | TATVAM-FAB18 | | | Animal | | India | | 2,480,954 | | 70 | 45 |
| GCF_047290855.1 | *W. cibaria* | | Sample-1 | | | Food | | India | | 2,540,597 | | 158 | 45 |
| GCF_048656945.1 | *W. cibaria* | | RAE-3 | | | Fermented Food | | China | | 1,818,703 | | 27 | 37 |
| GCF_049889075.1 | *W. cibaria* | | SDS2.1 | | | Unknown | | China | | 1,658,775 | | 9 | 43.5 |
| GCF_050586405.1 | *W. cibaria* | | JLK | | | Fermented Food | | Australia | | 2,277,843 | | 29 | 44.5 |
| GCF_050908175.1 | *W. cibaria* | | Xhu414 | | | Fermented Food | | China | | 2,358,664 | | 32 | 45 |
| GCF_902364765.1 | *W. cibaria* | | MGYG-HGUT-00248 | | | Human | | China | | 2,423,709 | | 3 | 45 |
| GCF_965137475.1 | *W. cibaria* | | CIP107376T | | | Unknown | | not provided | | 1,659,381 | | 1 | 39.5 |
| GCF_965138655.1 | *W. cibaria* | | CIP108011 | | | Unknown | | Unknown | | 2,332,950 | | 27 | 44.5 |
| GCF_000239955.1 | *W. confusa* | | LBAE C39-2 | | | Unknown | | Unknown | | 2,443,629 | | 72 | 44.5 |
| GCF_001436895.1 | *W. confusa* | | DSM 20196 | | | Fermented Food | | China | | 1,593,241 | | 1 | 41 |
| GCF_001884305.1 | *W. confusa* | | MBF8-1 | | | Food | | Indonesia | | 2,323,915 | | 56 | 44.5 |
| GCF_003284505.1 | *W. confusa* | | 32 | | | Animal | | Russia | | 2,262,214 | | 33 | 44.5 |
| GCF_003466065.1 | *W. confusa* | | AF03-13 | | | Human | | China | | 2,462,043 | | 145 | 44.5 |
| GCF_004766085.1 | *W. confusa* | | CCUG 37938 | | | Human | | Sweden | | 2,205,752 | | 42 | 44.5 |
| GCF_004766095.1 | *W. confusa* | | CCUG 39518 | | | Human | | Sweden | | 2,486,472 | | 28 | 45 |
| GCF_004766105.1 | *W. confusa* | | CCUG 36556 | | | Human | | Sweden | | 2,426,514 | | 40 | 45 |
| GCF_004766115.1 | *W. confusa* | | CCUG 51220 | | | Human | | Sweden | | 2,485,729 | | 40 | 44.5 |
| GCF_004766185.1 | *W. confusa* | | CCUG 30969 | | | Human | | Sweden | | 2,310,516 | | 30 | 44.5 |
| GCF_004766195.1 | *W. confusa* | | EJ61 | | | Food | | Finland | | 2,226,747 | | 45 | 44.5 |
| GCF_004766205.1 | *W. confusa* | | F71 | | | Food | | Finland | | 2,052,536 | | 2 | 38 |
| GCF_004766215.1 | *W. confusa* | | EJ12 | | | Food | | Finland | | 2,260,389 | | 47 | 44.5 |
| GCF_004766225.1 | *W. confusa* | | VTT E-163496 | | | Food | | Finland | | 2,161,388 | | 29 | 45 |
| GCF_004766265.1 | *W. confusa* | | VTT E-153460 | | | Food | | Finland | | 2,220,792 | | 54 | 45 |
| GCF_004766305.1 | *W. confusa* | | VTT E-072748 | | | Food | | Finland | | 2,198,240 | | 39 | 44.5 |
| GCF_004766325.1 | *W. confusa* | | EJ52 | | | Food | | Finland | | 2,235,316 | | 50 | 44.5 |
| GCF_004766335.1 | *W. confusa* | | CCUG 61560 | | | Human | | Sweden | | 2,280,085 | | 50 | 44.5 |
| GCF_004766355.1 | *W. confusa* | | VTT E-95616 | | | Fermented Food | | Finland | | 2,410,782 | | 56 | 44.5 |
| GCF_004766395.1 | *W. confusa* | | VTT E-143403 | | | Food | | Finland | | 2,279,882 | | 41 | 45 |
| GCF_004766405.1 | *W. confusa* | | VTT E-153457 | | | Food | | Finland | | 2,278,109 | | 45 | 44.5 |
| GCF_004766425.1 | *W. confusa* | | VTT E-90393 | | | Food | | Unknown | | 2,181,147 | | 30 | 45 |
| GCF_004771075.1 | *W. confusa* | | VTT E-133279 | | | Food | | India | | 2,244,220 | | 54 | 45 |
| GCF_007991875.1 | *W. confusa* | | NBRC 106469 | | | Food | | Nigeria | | 2,311,720 | | 42 | 45 |
| GCF_011044335.1 | *W. confusa* | | N17 | | | Animal | | not collected | | 2,292,343 | | 24 | 45 |
| GCF_014061025.1 | *W. confusa* | | CCK931 | | | Fermented Food | | South Korea | | 2,340,659 | | 82 | 44.5 |
| GCF_014694545.1 | *W. confusa* | | UC4052 | | | Unknown | | Italy | | 2,080,289 | | 88 | 45 |
| GCF_014845385.1 | *W. confusa* | | AP10A | | | Human | | India | | 2,244,313 | | 50 | 45 |
| GCF_015548055.1 | *W. confusa* | | 1001271B_151109_G12 | | | Human | | USA | | 2,167,360 | | 81 | 45 |
| GCF_015551765.1 | *W. confusa* | | 1001271B_151109_F1 | | | Human | | USA | | 2,147,793 | | 98 | 45 |
| GCF_015594935.1 | *W. confusa* | | WcL17 | | | Fermented Food | | Mexico | | 2,315,959 | | 52 | 44.5 |
| GCF_015594955.1 | *W. confusa* | | Wcp3a | | | Unknown | | Mexico | | 2,159,186 | | 78 | 45 |
| GCF_016465835.1 | *W. confusa* | | CCUG 61560 | | | Human | | Sweden | | 2,090,569 | | 91 | 45 |
| GCF_016465845.1 | *W. confusa* | | LMG 14040 | | | Animal | | Belgium | | 2,096,755 | | 113 | 45 |
| GCF_016465865.1 | *W. confusa* | | CCUG 54486 | | | Environment | | Sweden | | 2,271,866 | | 41 | 45 |
| GCF_016465875.1 | *W. confusa* | | CCUG 58143 | | | Human | | Sweden | | 2,285,793 | | 29 | 44.5 |
| GCF_016465895.1 | *W. confusa* | | CIP 102578 | | | Human | | France | | 2,335,379 | | 25 | 44.5 |
| GCF_016465935.1 | *W. confusa* | | CCUG 51220 | | | Human | | Sweden | | 2,291,289 | | 47 | 44.5 |
| GCF_016465945.1 | *W. confusa* | | CCUG 39518 | | | Human | | Sweden | | 2,302,034 | | 55 | 44.5 |
| GCF_016465965.1 | *W. confusa* | | CCUG 43002 | | | Human | | USA | | 2,338,194 | | 42 | 44.5 |
| GCF_016465985.1 | *W. confusa* | | CCUG 41966 | | | Human | | Belgium | | 2,338,394 | | 44 | 44.5 |
| GCF_016466005.1 | *W. confusa* | | CCUG 39957 | | | Human | | Sweden | | 2,359,005 | | 23 | 44.5 |
| GCF_016466065.1 | *W. confusa* | | CCUG 36556 | | | Human | | Sweden | | 2,330,935 | | 99 | 44.5 |
| GCF_016466085.1 | *W. confusa* | | CCUG 37938 | | | Human | | Sweden | | 2,338,112 | | 42 | 44.5 |
| GCF_016466095.1 | *W. confusa* | | CCUG 39150 | | | Human | | Sweden | | 2,149,009 | | 75 | 45 |
| GCF_016466115.1 | *W. confusa* | | CCUG 30969 | | | Human | | Sweden | | 2,170,823 | | 30 | 45 |
| GCF_016466155.1 | *W. confusa* | | CCUG 30943 | | | Human | | Sweden | | 2,385,778 | | 152 | 44.5 |
| GCF_016466175.1 | *W. confusa* | | CCUG 30763 | | | Animal | | Sweden | | 2,237,500 | | 30 | 45 |
| GCF_016466195.1 | *W. confusa* | | CCUG 17582 | | | Human | | Sweden | | 2,268,883 | | 44 | 45 |
| GCF_016466205.1 | *W. confusa* | | BCC 4255 | | | Fermented Food | | China | | 2,240,899 | | 42 | 45 |
| GCF_016466215.1 | *W. confusa* | | BCC 4230 | | | Fermented Food | | China | | 2,208,962 | | 37 | 44.5 |
| GCF_016466255.1 | *W. confusa* | | BCC 4194 | | | Fermented Food | | China | | 2,304,631 | | 55 | 45 |
| GCF_016466275.1 | *W. confusa* | | BCC 3262 | | | Fermented Food | | Poland | | 1,680,010 | | 15 | 40 |
| GCF_016466295.1 | *W. confusa* | | BCC 3038 | | | Fermented Food | | Poland | | 1,804,355 | | 34 | 39 |
| GCF_016466305.1 | *W. confusa* | | BCC 2344 | | | Fermented Food | | China | | 1,386,044 | | 1 | 40.5 |
| GCF_016466325.1 | *W. confusa* | | BCC 2384 | | | Fermented Food | | Poland | | 2,259,125 | | 36 | 44.5 |
| GCF_016466355.1 | *W. confusa* | | BCC 2330 | | | Fermented Food | | China | | 1,950,523 | | 31 | 38 |
| GCF_016466375.1 | *W. confusa* | | UC 748F4 | | | Fermented Food | | Italy | | 2,443,125 | | 34 | 45 |
| GCF_016466395.1 | *W. confusa* | | UC 710F16 | | | Fermented Food | | Italy | | 2,425,944 | | 27 | 45 |
| GCF_016466405.1 | *W. confusa* | | UC 724F13 | | | Fermented Food | | Italy | | 2,529,433 | | 2 | 44.5 |
| GCF_016466435.1 | *W. confusa* | | UC 048F3 | | | Fermented Food | | Italy | | 1,581,498 | | 18 | 41 |
| GCF_016466475.1 | *W. confusa* | | UC 024F6 | | | Fermented Food | | Italy | | 2,473,331 | | 22 | 45 |
| GCF_016466495.1 | *W. confusa* | | LMG 18478 | | | Fermented Food | | Malaysia | | 2,412,435 | | 41 | 45 |
| GCF_016466515.1 | *W. confusa* | | LMG 18476 | | | Fermented Food | | Malaysia | | 2,466,679 | | 42 | 45 |
| GCF_016466525.1 | *W. confusa* | | UC 010F3 | | | Fermented Food | | Italy | | 2,466,583 | | 36 | 45 |
| GCF_016466545.1 | *W. confusa* | | LMG 27187 | | | Fermented Food | | China | | 2,431,706 | | 38 | 45 |
| GCF_016466565.1 | *W. confusa* | | LMG 18477 | | | Fermented Food | | Malaysia | | 2,465,698 | | 39 | 45 |
| GCF_016466595.1 | *W. confusa* | | LMG 18475 | | | Fermented Food | | Malaysia | | 2,466,417 | | 40 | 45 |
| GCF_016466615.1 | *W. confusa* | | LMG 17705 | | | Food | | Malaysia | | 2,466,493 | | 34 | 45 |
| GCF_016466645.1 | *W. confusa* | | LMG 17695 | | | Food | | Malaysia | | 2,465,391 | | 34 | 45 |
| GCF_016466655.1 | *W. confusa* | | LMG 17698 | | | Food | | Malaysia | | 1,682,531 | | 38 | 41 |
| GCF_016466695.1 | *W. confusa* | | LMG 17696 | | | Food | | Malaysia | | 2,465,812 | | 39 | 45 |
| GCF_016466705.1 | *W. confusa* | | DSM 20194 | | | Food | | Italy | | 1,688,128 | | 42 | 41 |
| GCF_018916945.1 | *W. confusa* | | N65 | | | Animal | | China | | 2,427,357 | | 29 | 45 |
| GCF_019576155.1 | *W. confusa* | | LM1 | | | Animal | | China | | 2,522,263 | | 6 | 45 |
| GCF_024505545.1 | *W. confusa* | | B5 | | | Animal | | USA | | 2,368,433 | | 3 | 45 |
| GCF_024505585.1 | *W. confusa* | | L1 | | | Human | | USA | | 1,673,321 | | 30 | 43.5 |
| GCF_025127115.1 | *W. confusa* | | 1521 | | | Fermented Food | | Cote d'Ivoire | | 2,534,886 | | 4 | 45 |
| GCF_025127135.1 | *W. confusa* | | 1432 | | | Fermented Food | | Mali | | 2,597,955 | | 7 | 44.5 |
| GCF_025127195.1 | *W. confusa* | | 1310 | | | Fermented Food | | Cote d'Ivoire | | 1,639,510 | | 33 | 39 |
| GCF_025128995.1 | *W. confusa* | | 1477 | | | Fermented Food | | Kenya | | 1,682,559 | | 26 | 40 |
| GCF_025129015.1 | *W. confusa* | | 1523 | | | Fermented Food | | Cote d'Ivoire | | 1,750,755 | | 35 | 38.5 |
| GCF_025129055.1 | *W. confusa* | | 1474 | | | Fermented Food | | Kenya | | 1,907,411 | | 47 | 38 |
| GCF_025129065.1 | *W. confusa* | | 1479 | | | Fermented Food | | Somalia | | 1,908,066 | | 43 | 38 |
| GCF_025129095.1 | *W. confusa* | | 1433 | | | Fermented Food | | Mali | | 1,953,096 | | 50 | 38 |
| GCF_025129115.1 | *W. confusa* | | 1407 | | | Fermented Food | | Mali | | 1,953,316 | | 48 | 38 |
| GCF_025129135.1 | *W. confusa* | | 1316 | | | Fermented Food | | Cote d'Ivoire | | 1,953,167 | | 46 | 38 |
| GCF_025132785.1 | *W. confusa* | | 1486 | | | Food | | Somalia | | 1,952,675 | | 51 | 38 |
| GCF_025186425.1 | *W. confusa* | | CIRM-BIA 2234 | | | Fermented Food | | France | | 1,953,585 | | 49 | 38 |
| GCF_025395355.1 | *W. confusa* | | 44 | | | Food | | Australia | | 1,953,114 | | 49 | 38 |
| GCF_025395365.1 | *W. confusa* | | 744 | | | Food | | Australia | | 2,218,368 | | 56 | 44.5 |
| GCF_025960385.1 | *W. confusa* | | WiKim51 | | | Fermented Food | | South Korea | | 2,252,685 | | 141 | 44.5 |
| GCF_027658705.1 | *W. confusa* | | AM109-41 | | | Human | | China | | 2,421,541 | | 36 | 45 |
| GCF_027675005.1 | *W. confusa* | | AM110-56 | | | Human | | China | | 1,789,774 | | 18 | 36.5 |
| GCF_027675445.1 | *W. confusa* | | AM110-142 | | | Human | | China | | 2,494,306 | | 185 | 45 |
| GCF_027677765.1 | *W. confusa* | | TM115-80 | | | Human | | China | | 2,398,927 | | 35 | 45 |
| GCF_027723565.1 | *W. confusa* | | AM110-04 | | | Human | | China | | 2,401,801 | | 36 | 45 |
| GCF_027723625.1 | *W. confusa* | | AM110-103 | | | Human | | China | | 2,084,869 | | 82 | 38 |
| GCF_027723725.1 | *W. confusa* | | AM110-108 | | | Human | | China | | 2,083,985 | | 87 | 38 |
| GCF_027945635.1 | *W. confusa* | | 10D | | | Food | | USA | | 2,255,678 | | 54 | 44.5 |
| GCF_027945675.1 | *W. confusa* | | 10E | | | Food | | USA | | 2,243,340 | | 55 | 44.5 |
| GCF_029536205.1 | *W. confusa* | | CYLB30 | | | Fermented Food | | Taiwan | | 2,284,356 | | 59 | 45 |
| GCF_030939325.1 | *W. confusa* | | NN 1 | | | Human | | Bulgaria | | 2,433,122 | | 56 | 45 |
| GCF_030939515.1 | *W. confusa* | | AG 2-6 | | | Human | | Bulgaria | | 2,410,643 | | 90 | 45 |
| GCF_034083425.1 | *W. confusa* | | YTU8 | | | Fermented Food | | Turkey | | 2,082,822 | | 86 | 38 |
| GCF_034083535.1 | *W. confusa* | | YTU17 | | | Fermented Food | | Turkey | | 2,269,536 | | 67 | 44.5 |
| GCF_034083545.1 | *W. confusa* | | YTU2 | | | Fermented Food | | Turkey | | 2,374,653 | | 61 | 44.5 |
| GCF_036224425.1 | *W. confusa* | | B-333 | | | Unknown | | Unknown | | 2,414,889 | | 128 | 44.5 |
| GCF_036430945.1 | *W. confusa* | | FI11592 | | | Fermented Food | | Ghana | | 2,386,050 | | 64 | 44.5 |
| GCF_040705115.1 | *W. confusa* | | PM7 | | | Human | | Cameroon | | 2,242,696 | | 51 | 44.5 |
| GCF_041346935.1 | *W. confusa* | | HYF86 | | | Food | | China | | 2,271,082 | | 58 | 44.5 |
| GCF_041729635.1 | *W. confusa* | | SY628 | | | Fermented Food | | China | | 2,218,392 | | 76 | 44.5 |
| GCF_043558845.1 | *W. confusa* | | ProY_51 | | | Food | | Bangladesh | | 2,044,699 | | 49 | 38 |
| GCF_046558495.1 | *W. confusa* | | W1 | | | Human | | India | | 2,378,296 | | 107 | 44.5 |
| GCF_046600975.1 | *W. confusa* | | mrbd | | | Fermented Food | | Bangladesh | | 2,418,677 | | 60 | 45 |
| GCF_051844195.1 | *W. confusa* | | IB | | | Unknown | | Brazil | | 2,221,878 | | 50 | 45 |
| GCF_900166935.1 | *W. confusa* | | AB3E41 | | | Fermented Food | | Cote d'Ivoire | | 1,692,694 | | 24 | 43.5 |
| GCF_902363865.1 | *W. confusa* | | MGYG-HGUT-00155 | | | Human | | China | | 2,485,334 | | 52 | 45 |
| GCF_965135865.1 | *W. confusa* | | CIP103172T | | | Unknown | | Unknown | | 2,284,682 | | 45 | 44.5 |
| GCF_014397255.1 | *W. diestrammenae* | | KACC 16890 | | | Animal | | South Korea | | 2,294,783 | | 19 | 45 |
| GCF_023657305.1 | *W. diestrammenae* | | DSM 27840 | | | Animal | | South Korea | | 2,442,686 | | 50 | 45 |
| GCF_025908565.2 | *W. fermenti* | | BK2 | | | Fermented Food | | South Korea | | 2,428,300 | | 34 | 45 |
| GCF_040137825.1 | | *W. fermenti* | | GP1 | Human | | Uganda | | 2,252,621 | | 44 | | 44.5 |
| GCF_016466635.1 | *W. fermenti* | | LMG 11983 | | | Fermented Food | | Unknown | | 2,473,856 | | 47 | 45 |
| GCF_004771295.1 | *W. fermenti* | | VTT E-90392 | | | Food | | Finland | | 2,250,519 | | 38 | 45 |
| GCF_016466735.1 | *W. fermenti* | | DSM 20196 | | | Food | | Italy | | 2,466,044 | | 30 | 45 |
| GCF_046489095.1 | *W. fermenti* | | 36 | | | Food | | Australia | | 2,455,430 | | 111 | 44.5 |
| GCF_027679765.1 | *W. fermenti* | | TM115-115 | | | Human | | China | | 2,397,717 | | 35 | 45 |
| GCF_009906195.1 | *W. fermenti* | | 718955 | | | Animal | | USA | | 2,333,989 | | 48 | 44.5 |
| GCF_044068395.1 | *W. fermenti* | | GIR46L4 | | | Animal | | Brazil | | 2,383,987 | | 43 | 45 |
|  |  | |  | | |  | |  | |  | |  |  |
| GCF_000420365.1 | *W. halotolerans* | | DSM 20190 | | | Fermented Food | | Unknown | | 2,463,493 | | 28 | 45 |
| GCF_001436865.1 | *W. halotolerans* | | DSM 20190 | | | Fermented Food | | China | | 2,497,578 | | 5 | 44.5 |
| GCF_005405545.1 | *W. hellenica* | | NBRC 15553 | | | Fermented Food | | Greece | | 2,332,701 | | 2 | 45 |
| GCF_006540065.1 | *W. hellenica* | | NBRC 15553 | | | Fermented Food | | Unknown | | 2,354,450 | | 36 | 44.5 |
| GCF_012396435.1 | *W. hellenica* | | CCUG 33494 | | | Fermented Food | | missing | | 2,408,088 | | 43 | 45 |
| GCF_045527605.1 | *W. hellenica* | | FAM 25332 | | | Fermented Food | | Switzerland | | 2,437,073 | | 33 | 45 |
| GCF_045527645.1 | *W. hellenica* | | FAM 25330 | | | Fermented Food | | Switzerland | | 2,530,296 | | 33 | 44.5 |
| GCF_900095015.1 | *W. hellenica* | | R-53116 | | | Unknown | | Unknown | | 2,267,717 | | 46 | 45 |
| GCF_965140135.1 | *W. hellenica* | | CIP109879T | | | Unknown | | Unknown | | 2,288,038 | | 51 | 45 |
| GCF_001932615.1 | *W. jogaejeotgali* | | FOL01 | | | Fermented Food | | South Korea | | 2,142,331 | | 33 | 45 |
| GCF_001437425.1 | *W. minor* | | DSM 20014 | | | Fermented Food | | China | | 2,462,405 | | 40 | 45 |
| GCF_018257015.1 | *W. minor* | | Dm-2019-34 | | | Animal | | USA | | 2,243,237 | | 45 | 45 |
| GCF_965196875.1 | *W. minor* | | R-84915 | | | Animal | | France | | 1,572,640 | | 19 | 41 |
| GCF_000691805.2 | *W. oryzae* | | SG25 | | | Fermented Food | | Japan | | 2,448,243 | | 21 | 45 |
| GCF_000160575.1 | *W. paramesenteroides* | | ATCC 33313 | | | Human | | USA | | 2,230,749 | | 57 | 44.5 |
| GCF_002386265.1 | *W. paramesenteroides* | | FDAARGOS_414 | | | Environment | | Unknown | | 2,265,425 | | 41 | 45 |
| GCF_004214915.1 | *W. paramesenteroides* | | WpK4 | | | Animal | | Brazil | | 2,267,706 | | 35 | 44.5 |
| GCF_006406215.1 | *W. paramesenteroides* | | TR212 | | | Fermented Food | | Ireland | | 2,287,257 | | 155 | 44.5 |
| GCF_008689905.1 | *W. paramesenteroides* | | DmW_115114 | | | Animal | | USA | | 2,225,995 | | 139 | 44.5 |
| GCF_008689955.1 | *W. paramesenteroides* | | DmW_115113 | | | Animal | | USA | | 2,406,256 | | 1 | 45 |
| GCF_008689965.1 | *W. paramesenteroides* | | DmW_115112 | | | Animal | | USA | | 2,406,256 | | 1 | 45 |
| GCF_008689975.1 | *W. paramesenteroides* | | DmW_115 | | | Animal | | USA | | 2,024,293 | | 1 | 38 |
| GCF_008690005.1 | *W. paramesenteroides* | | DmW_115116 | | | Animal | | USA | | 1,998,272 | | 12 | 38 |
| GCF_008690035.1 | *W. paramesenteroides* | | DmW_109 | | | Animal | | USA | | 1,915,948 | | 19 | 38 |
| GCF_008690065.1 | *W. paramesenteroides* | | DmW_107104 | | | Animal | | USA | | 2,114,173 | | 35 | 38 |
| GCF_008690075.1 | *W. paramesenteroides* | | DmW_107108 | | | Animal | | USA | | 2,089,458 | | 35 | 38 |
| GCF_008690085.1 | *W. paramesenteroides* | | DmW_107105 | | | Animal | | USA | | 2,110,401 | | 36 | 38 |
| GCF_008690135.1 | *W. paramesenteroides* | | DmW_107101 | | | Animal | | USA | | 2,511,021 | | 7 | 44.5 |
| GCF_008690155.1 | *W. paramesenteroides* | | DmW_107102 | | | Animal | | USA | | 2,457,505 | | 32 | 45 |
| GCF_008690165.1 | *W. paramesenteroides* | | DmW_107106 | | | Animal | | USA | | 2,033,487 | | 2 | 38 |
| GCF_008690175.1 | *W. paramesenteroides* | | DmW_107100 | | | Animal | | USA | | 2,439,530 | | 23 | 45 |
| GCF_008690185.1 | *W. paramesenteroides* | | DmW_107 | | | Animal | | USA | | 1,593,258 | | 1 | 41 |
| GCF_008690195.1 | *W. paramesenteroides* | | DmW_107099 | | | Animal | | USA | | 2,461,013 | | 50 | 45 |
| GCF_011009575.1 | *W. paramesenteroides* | | N43 | | | Animal | | United Kingdom | | 2,455,505 | | 42 | 45 |
| GCF_011009625.1 | *W. paramesenteroides* | | A37 | | | Animal | | United Kingdom | | 2,235,757 | | 18 | 44.5 |
| GCF_015689215.1 | *W. paramesenteroides* | | STCH-BD1 | | | Fermented Food | | USA | | 2,165,802 | | 43 | 45 |
| GCF_018991665.1 | *W. paramesenteroides* | | 3.2.24 | | | Fermented Food | | USA | | 2,391,331 | | 59 | 45 |
| GCF_023740595.1 | *W. paramesenteroides* | | DRD-142 | | | Fermented Food | | Greece | | 2,540,602 | | 155 | 44.5 |
| GCF_023740605.1 | *W. paramesenteroides* | | DRD-169 | | | Fermented Food | | Greece | | 2,244,559 | | 49 | 44.5 |
| GCF_023740635.1 | *W. paramesenteroides* | | DRD-172 | | | Fermented Food | | Greece | | 2,336,244 | | 73 | 44.5 |
| GCF_023740655.1 | *W. paramesenteroides* | | DRD-194 | | | Fermented Food | | Greece | | 2,470,124 | | 57 | 45 |
| GCF_023740695.1 | *W. paramesenteroides* | | DRD-187 | | | Fermented Food | | Greece | | 2,280,122 | | 56 | 44.5 |
| GCF_023740715.1 | *W. paramesenteroides* | | DRD-149 | | | Fermented Food | | Greece | | 2,007,410 | | 2 | 38 |
| GCF_023740775.1 | *W. paramesenteroides* | | DRD-105 | | | Food | | Greece | | 2,200,812 | | 51 | 44.5 |
| GCF_023740795.1 | *W. paramesenteroides* | | DRD-137 | | | Animal | | Greece | | 2,458,354 | | 55 | 45 |
| GCF_023744335.1 | *W. paramesenteroides* | | DRD-39 | | | Fermented Food | | Greece | | 2,416,428 | | 132 | 44.5 |
| GCF_025127055.1 | *W. paramesenteroides* | | 1398 | | | Fermented Food | | Mali | | 2,401,522 | | 4 | 45 |
| GCF_025127095.1 | *W. paramesenteroides* | | 1396 | | | Fermented Food | | Mali | | 2,335,357 | | 29 | 44.5 |
| GCF_025128975.1 | *W. paramesenteroides* | | 1397 | | | Fermented Food | | Mali | | 1,946,180 | | 9 | 38 |
| GCF_025131195.1 | *W. paramesenteroides* | | 1402 | | | Animal | | Mali | | 2,465,329 | | 9 | 45 |
| GCF_028994215.1 | *W. paramesenteroides* | | FL3 | | | Fermented Food | | China | | 2,499,177 | | 7 | 45 |
| GCF_029450415.1 | *W. paramesenteroides* | | B1 | | | Animal | | Finland | | 2,503,562 | | 3 | 45 |
| GCF_029450425.1 | *W. paramesenteroides* | | C8 | | | Animal | | Finland | | 1,940,694 | | 2 | 37 |
| GCF_029450455.1 | *W. paramesenteroides* | | SJ21 | | | Animal | | Finland | | 2,264,616 | | 3 | 45 |
| GCF_029450465.1 | *W. paramesenteroides* | | SJ27-5 | | | Animal | | Finland | | 1,658,031 | | 2 | 41 |
| GCF_029450475.1 | *W. paramesenteroides* | | SJ27-4 | | | Animal | | Finland | | 2,339,215 | | 4 | 44.5 |
| GCF_030168775.1 | *W. paramesenteroides* | | W31 | | | Animal | | Finland | | 2,350,892 | | 3 | 45 |
| GCF_036327715.1 | *W. paramesenteroides* | | MbWp-142 | | | Fermented Food | | China | | 2,518,000 | | 4 | 45 |
| GCF_041147055.1 | *W. paramesenteroides* | | 3.8.13 | | | Fermented Food | | USA | | 2,438,900 | | 1 | 45 |
| GCF_044068415.1 | *W. paramesenteroides* | | V3B-11 | | | Animal | | Brazil | | 1,927,134 | | 25 | 36.5 |
| GCF_045005335.1 | *W. paramesenteroides* | | JL-5 | | | Fermented Food | | China | | 2,418,986 | | 1 | 45 |
| GCF_045006675.1 | *W. paramesenteroides* | | LCW-28 | | | Fermented Food | | China | | 2,242,023 | | 40 | 45 |
| GCF_046588255.1 | *W. paramesenteroides* | | TATVAM-FAB90 | | | Animal | | India | | 2,115,109 | | 54 | 45 |
| GCF_050613345.1 | *W. paramesenteroides* | | A47_1 | | | Animal | | China | | 2,458,858 | | 10 | 45 |
| GCF_051370585.1 | *W. paramesenteroides* | | HC970 | | | Animal | | China | | 1,995,719 | | 3 | 38 |
| GCF_051370695.1 | *W. paramesenteroides* | | HC968 | | | Animal | | China | | 1,963,850 | | 1 | 38.5 |
| GCF_051371825.1 | *W. paramesenteroides* | | HC330 | | | Animal | | China | | 1,990,966 | | 3 | 38 |
| GCF_051903945.1 | *W. paramesenteroides* | | DSM 20288 | | | Fermented Food | | China | | 1,927,724 | | 70 | 37 |
| GCF_902374075.1 | *W. paramesenteroides* | | MGYG-HGUT-01340 | | | Human | | Unknown | | 1,924,263 | | 55 | 37 |
| GCF_964186645.1 | *W. paramesenteroides* | | UFTM 2.6.1 | | | Animal | | Brazil | | 2,489,354 | | 47 | 45 |
| GCF_005405285.1 | *W. sagaensis* | | X0750 | | | Fermented Food | | China | | 2,280,991 | | 74 | 45 |
| GCF_010306265.1 | *W. sagaensis* | | X0278 | | | Fermented Food | | China | | 2,281,401 | | 30 | 44.5 |
| GCF_010306445.1 | *W. sagaensis* | | X0401 | | | Fermented Food | | China | | 2,060,860 | | 61 | 38 |
| GCF_024623405.1 | *W. sagaensis* | | HY604 | | | Animal | | China | | 2,436,776 | | 23 | 45 |
| GCF_041281755.1 | *W. sagaensis* | | ZD-66 | | | Fermented Food | | China | | 2,467,975 | | 28 | 45 |
| GCF_042659285.1 | *W. sagaensis* | | CCM 8924 | | | Unknown | | Unknown | | 2,250,349 | | 32 | 45 |
| GCF_001761545.1 | *W. soli* | | KACC 11848 | | | Fermented Food | | South Korea | | 2,427,098 | | 19 | 45 |
| GCF_003353445.1 | *W. soli* | | CECT 7031 | | | Unknown | | USA | | 2,507,636 | | 29 | 45 |
| GCF_007954545.1 | *W. soli* | | CBA3633 | | | Fermented Food | | South Korea | | 1,678,388 | | 1 | 44 |
| GCF_007991555.1 | *W. soli* | | NBRC 106074 | | | Environment | | Sweden | | 2,472,619 | | 3 | 45 |
| GCF_012396465.1 | *W. soli* | | CCUG 46608 | | | Environment | | Sweden | | 2,530,145 | | 55 | 44.5 |
| GCF_021604945.1 | *W. soli* | | DB-2 | | | Fermented Food | | Japan | | 2,080,060 | | 2 | 38 |
| GCF_025395255.1 | *W. soli* | | 498 | | | Food | | Australia | | 2,343,022 | | 1 | 45.5 |
| GCF_049668835.1 | *W. soli* | | 3-88. | | | Environment | | Iceland | | 1,449,263 | | 1 | 40.5 |
| GCF_000732905.1 | *W. tructae* | | WS08 | | | Animal | | Brazil | | 2,072,893 | | 67 | 38 |
| GCF_018502505.1 | *W. tructae* | | W-1 | | | Animal | | Mexico | | 2,106,512 | | 38 | 38 |
| GCF_000750515.1 | *W. tructae* | | WS74 | | | Animal | | Brazil | | 1,980,223 | | 36 | 38 |
| GCF_000750535.1 | *W. tructae* | | WS105 | | | Animal | | Brazil | | 2,317,857 | | 72 | 45 |
| GCF_000320345.1 | *W. tructae* | | NC36 | | | Animal | | USA | | 2,284,024 | | 71 | 45 |
| GCF_050942155.1 | *W. tructae* | | TB02 | | | Animal | | Viet Nam | | 2,122,255 | | 45 | 37.5 |
| GCF_016908785.1 | *W. uvarum* | | DSM 28060 | | | Unknown | | Unknown | | 2,294,431 | | 39 | 44.5 |
| GCF_023657405.1 | *W. uvarum* | | B18NM42 | | | Food | | Greece | | 2,149,463 | | 4 | 38 |
| GCF_001437355.1 | *W. viridescens* | | DSM 20410 | | | Unknown | | China | | 1,626,940 | | 1 | 41 |
| GCF_006538805.1 | *W. viridescens* | | NBRC 3949 | | | Unknown | | Unknown | | 2,258,481 | | 54 | 45 |
| GCF_014789145.1 | *W. viridescens* | | NJ100 | | | Food | | China | | 2,346,332 | | 38 | 44.5 |
| GCF_019641425.1 | *W. viridescens* | | LB10G | | | Fermented Food | | Denmark | | 2,506,353 | | 21 | 45 |
| GCF_020553585.1 | *W. viridescens* | | MSK.22.110 | | | Human | | USA | | 1,980,223 | | 36 | 38 |
| GCF_020553625.1 | *W. viridescens* | | MSK.22.125 | | | Human | | USA | | 2,019,488 | | 26 | 38 |
| GCF_027692285.1 | *W. viridescens* | | OF45-4pH10A | | | Human | | China | | 2,224,255 | | 32 | 44.5 |
| GCF_030323345.1 | *W. viridescens* | | PC45 | | | Animal | | Pakistan | | 2,322,316 | | 29 | 45 |
| GCF_041379745.1 | *W. viridescens* | | XBS25 | | | Fermented Food | | China | | 2,454,708 | | 36 | 45 |
| GCF_052783125.1 | *W. viridescens* | | HDY-8 | | | Fermented Food | | Thailand | | 1,815,893 | | 36 | 37 |
| GCF_965196855.1 | *W. viridescens* | | LMG 30253 | | | Animal | | Belgium | | 1,775,323 | | 15 | 41 |
| GCF_041871065.1 | *Weissella* sp. | | JLP1 | | | Animal | | China | | 2,406,530 | | 50 | 45 |
| GCF_004766315.1 | *Weissella* sp. | | VTT E-062653 | | | Fermented Food | | Finland | | 2,260,674 | | 49 | 44.5 |
| GCF_004521965.1 | *Weissella* sp. | | SP7 | | | Animal | | Kuwait | | 2,342,914 | | 1 | 45.5 |
| GCF_003862435.1 | *Weissella* sp. | | UCO-SMC3 | | | Animal | | Chile | | 1,816,487 | | 33 | 37 |
| GCF_045799135.1 | *Weissella* sp. | | MSCH1 | | | Food | | India | | 1,810,593 | | 26 | 39 |

**Table S2** Summarize genome numbers in each *Weissella* species.

| Species | Genome count |
| --- | --- |
| *Weissella confusa* | 119 |
| *Weissella cibaria* | 109 |
| *Weissella paramesenteroides* | 56 |
| *Weissella viridescens* | 11 |
| *Weissella fermenti* | 9 |
| *Weissella soli* | 8 |
| *Weissella hellenica* | 7 |
| *Weissella sagaensis* | 6 |
| *Weissella tructae* | 6 |
| *Weissella minor* | 3 |
| *Weissella uvarum* | 2 |
| *Weissella halotolerans* | 2 |
| *Weissella diestrammenae* | 2 |
| *Weissella oryzae* | 1 |
| *Weissella jogaejeotgali* | 1 |
| *Weissella* sp. | 5 |

**Table S3** Summary of EPS synteny subgroups identified in *Weissella*.

| Synteny sub-group | Number of clusters | Species |
| --- | --- | --- |
| A | 4 | *W. confusa* (4) |
| B | 124 | *W. confusa* (119)*, W. cibaria* (4), *W. fermenti* (1) |
| C | 20 | *W. cibaria* (17)*, W. confusa* (2)*,* *W. paramesenteroides* (1) |
| D | 88 | *W. cibaria* (87), *Weissella* sp. (1) |
| E | 4 | *W. cibaria* (4) |
| F | 43 | *W. confusa* (36)*, W. cibaria* (5), *W. paramesenteroides* (2) |

**Table S4** Putative probiotic-associated genetic markers used for functional category screening and prevalence found in *Weissella* genomes.

| Category | Gene | Gene description | Prevalence found in species |
| --- | --- | --- | --- |
| GIT tolerance | *cfa* | Cyclopropane-fatty-acyl-phospholipid synthase; modifies membrane fatty acids and may support acid or membrane stress tolerance. | *W. confusa* 119/119 (100.0%); *W. fermenti* 9/9 (100.0%); *Weissella* sp. 2/5 (40.0%) |
|  | *arcA* | Arginine deiminase; part of the arginine deiminase pathway associated with acid stress adaptation. | *W. confusa* 119/119 (100.0%); *W. cibaria* 109/109 (100.0%); *W. fermenti* 9/9 (100.0%); *Weissella* sp. 3/5 (60.0%) |
|  | *arcB* | Ornithine carbamoyltransferase; arginine deiminase pathway enzyme involved in arginine catabolism. | *W. confusa* 119/119 (100.0%); *W. tructae* 6/6 (100.0%); *Weissella* sp. 1/5 (20.0%) |
|  | *atpA-H* | F0F1 ATP synthase subunits; proton-translocating ATPase complex associated with intracellular pH homeostasis and acid tolerance. | *W. confusa* 119/119 (100.0%); *W. cibaria* 109/109 (100.0%); *W. fermenti* 9/9 (100.0%); *W. soli* 8/8 (100.0%); *W. tructae* 6/6 (100.0%); *Weissella* sp. 4/5 (80.0%) |
|  | *clcA* | Chloride/proton antiporter; contributes to chloride and proton homeostasis. | *W. confusa* 119/119 (100.0%); *W. cibaria* 109/109 (100.0%); *W. paramesenteroides* 2/56 (3.6%); *W. fermenti* 9/9 (100.0%); *W. sagaensis* 1/6 (16.7%); *Weissella* sp. 4/5 (80.0%); *W. jogaejeotgali* 1/1 (100.0%) |
|  | *dltA-D* | D-alanylation pathway for teichoic acids; cell envelope modification associated with acid, bile, and cell-envelope stress tolerance. | *W. confusa* 116/119 (97.5%); *W. fermenti* 9/9 (100.0%); *W. tructae* 6/6 (100.0%); *Weissella* sp. 2/5 (40.0%) |
|  | *nhaC* | Sodium/proton antiporter; contributes to ion and pH homeostasis. | *W. confusa* 119/119 (100.0%); *W. cibaria* 109/109 (100.0%); *W. paramesenteroides* 56/56 (100.0%); *W. fermenti* 9/9 (100.0%); W. hellenica 7/7 (100.0%); *W. sagaensis* 6/6 (100.0%); *Weissella* sp. 4/5 (80.0%); *W. jogaejeotgali* 1/1 (100.0%) |
|  | *lmrA* | ABC transporter; contribute to tolerance against bile. | *W. confusa* 119/119 (100.0%); *W. cibaria* 109/109 (100.0%); *W. fermenti* 9/9 (100.0%); *Weissella* sp. 3/5 (60.0%) |
| Adhesion/colonization | *tuf* | Elongation factor Tu; translation factor with reported moonlighting surface-adhesion roles in some bacteria. | *W. confusa* 119/119 (100.0%); *W. cibaria* 109/109 (100.0%); *W. paramesenteroides* 56/56 (100.0%); *W. fermenti* 9/9 (100.0%); *W. hellenica* 7/7 (100.0%); *W. sagaensis* 6/6 (100.0%); *Weissella* sp. 4/5 (80.0%); *W. jogaejeotgali* 1/1 (100.0%) |
|  | *gap* | Glyceraldehyde-3-phosphate dehydrogenase; metabolic enzyme with reported moonlighting adhesion roles in some bacteria. | *W. confusa* 119/119 (100.0%); *W. cibaria* 109/109 (100.0%); *W. paramesenteroides* 56/56 (100.0%); *W. fermenti* 9/9 (100.0%); W. soli 8/8 (100.0%); *W. hellenica* 7/7 (100.0%); *W. sagaensis* 6/6 (100.0%); *Weissella* sp. 4/5 (80.0%); *W. jogaejeotgali* 1/1 (100.0%) |
|  | *fimA* | Fimbrial protein; surface attachment. | *W. confusa* 119/119 (100.0%); *W. cibaria* 109/109 (100.0%); *W. fermenti* 9/9 (100.0%); *Weissella* sp. 4/5 (80.0%) |
|  | *icaA-D* | Intercellular adhesion/biofilm-associated genes involved in polysaccharide adhesin or biofilm matrix biosynthesis in some bacteria. | *W. confusa* 119/119 (100.0%); *W. viridescens* 11/11 (100.0%); *W. fermenti* 9/9 (100.0%); *Weissella* sp. 2/5 (40.0%) |
|  | *cna* | Collagen-binding adhesin; putative marker for interaction with host extracellular matrix. | *W. viridescens* 11/11 (100.0%); *Weissella* sp. 1/5 (20.0%) |
| General stress resistance | *grpE* | Nucleotide-exchange factor for DnaK; heat-shock chaperone system component. | *W. confusa* 119/119 (100.0%); *W. cibaria* 109/109 (100.0%); *W. fermenti* 9/9 (100.0%); *W. hellenica* 7/7 (100.0%); *W. sagaensis* 6/6 (100.0%); *Weissella* sp. 4/5 (80.0%) |
|  | *clpE* | ATP-dependent Clp protease ATP-binding subunit; stress response and protein quality control. | *W. cibaria* 109/109 (100.0%); *W. soli* 8/8 (100.0%); *Weissella* sp. 2/5 (40.0%) |
|  | *clpL* | ATP-dependent Clp ATPase; associated with heat and general stress adaptation. | *W. paramesenteroides* 13/56 (23.2%); *W. sagaensis* 5/6 (83.3%); *W. jogaejeotgali* 1/1 (100.0%) |
|  | *clpP* | ATP-dependent Clp protease proteolytic subunit; removes damaged proteins under stress. | *W. paramesenteroides* 56/56 (100.0%); *W. viridescens* 11/11 (100.0%); *W. hellenica* 7/7 (100.0%); *W. sagaensis* 6/6 (100.0%); *W. tructae* 6/6 (100.0%); *Weissella* sp. 1/5 (20.0%); *W. jogaejeotgali* 1/1 (100.0%) |
|  | *clpX* | ATP-dependent Clp protease ATP-binding subunit; protein quality control and stress response. | *W. confusa* 119/119 (100.0%); *W. cibaria* 109/109 (100.0%); *W. paramesenteroides* 56/56 (100.0%); *W. fermenti* 9/9 (100.0%); *W. hellenica* 7/7 (100.0%); *W. sagaensis* 6/6 (100.0%); *W. tructae* 6/6 (100.0%); *Weissella* sp. 4/5 (80.0%); *W. jogaejeotgali* 1/1 (100.0%) |
|  | *ftsH* | Membrane-associated ATP-dependent metalloprotease; protein quality control and membrane stress response. | *W. cibaria* 109/109 (100.0%); *W. paramesenteroides* 56/56 (100.0%); *W. tructae* 6/6 (100.0%); *Weissella* sp. 2/5 (40.0%) |
|  | *hrcA* | Heat-inducible transcriptional repressor; regulates heat-shock genes. | *W. confusa* 119/119 (100.0%); *W. cibaria* 109/109 (100.0%); *W. fermenti* 9/9 (100.0%); W. hellenica 7/7 (100.0%); *W. sagaensis* 6/6 (100.0%); *Weissella* sp. 4/5 (80.0%) |
|  | *cspA-C* | Cold-shock proteins; adaptation to low temperature and environmental stress. | *W. paramesenteroides* 56/56 (100.0%); *W. viridescens* 11/11 (100.0%); *W. soli* 8/8 (100.0%); *W. hellenica* 7/7 (100.0%); *W. sagaensis* 6/6 (100.0%); *Weissella* sp. 1/5 (20.0%) |
|  | *mscL* | Large-conductance mechanosensitive channel; osmotic down-shock response. | *W. confusa* 119/119 (100.0%); *W. cibaria* 109/109 (100.0%); *W. paramesenteroides* 56/56 (100.0%); *W. fermenti* 9/9 (100.0%); *W.* hellenica 7/7 (100.0%); *W. sagaensis* 6/6 (100.0%); *W. tructae* 6/6 (100.0%); *Weissella* sp. 4/5 (80.0%); *W. jogaejeotgali* 1/1 (100.0%) |
|  | *proX* | Glycine betaine/proline ABC transporter substrate-binding protein. | *W. confusa* 95/119 (79.8%); *W. cibaria* 1/109 (0.9%); *W. fermenti* 4/9 (44.4%); *Weissella* sp. 1/5 (20.0%) |
|  | *treB* | Trehalose-specific PTS transporter component; trehalose uptake/metabolism associated with stress protection. | *W. confusa* 119/119 (100.0%); *W. fermenti* 9/9 (100.0%); *Weissella* sp. 2/5 (40.0%) |
| Oxidative stress resistance | *trxA* | Thioredoxin; redox-active protein involved in oxidative stress defense. | *W. confusa* 119/119 (100.0%); *W. cibaria* 109/109 (100.0%); *W. paramesenteroides* 56/56 (100.0%); *W. viridescens* 11/11 (100.0%); *W.* fermenti 9/9 (100.0%); *W. hellenica* 7/7 (100.0%); *W. sagaensis* 6/6 (100.0%); *Weissella* sp. 5/5 (100.0%); *W. minor* 3/3 (100.0%); *W. uvarum* 2/2 (100.0%); *W. jogaejeotgali* 1/1 (100.0%) |
|  | *trxB* | Thioredoxin reductase; maintains reduced thioredoxin and cellular redox balance. | *W. confusa* 119/119 (100.0%); *W. cibaria* 109/109 (100.0%); *W. paramesenteroides* 56/56 (100.0%); *W. viridescens* 11/11 (100.0%); *W. fermenti* 9/9 (100.0%); *W. hellenica* 7/7 (100.0%); *W. sagaensis* 6/6 (100.0%); *Weissella* sp. 5/5 (100.0%); *W. uvarum* 2/2 (100.0%); *W. jogaejeotgali* 1/1 (100.0%) |
|  | *gor* | Glutathione reductase-like enzyme; redox homeostasis marker. | *W. confusa* 119/119 (100.0%); *W. cibaria* 109/109 (100.0%); *W. paramesenteroides* 56/56 (100.0%); *W. fermenti* 9/9 (100.0%); *W. soli* 8/8 (100.0%); *Weissella* sp. 4/5 (80.0%); *W. jogaejeotgali* 1/1 (100.0%) |
|  | *nox* | NADH oxidase; contributes to oxygen/redox balance. | *W. confusa* 118/119 (99.2%); *W. cibaria* 109/109 (100.0%); *W. viridescens* 11/11 (100.0%); *W.* *fermenti* 9/9 (100.0%); *Weissella* sp. 4/5 (80.0%) |
|  | *perR* | Peroxide stress regulator; regulates oxidative stress response genes. | *W. paramesenteroides* 56/56 (100.0%) |
|  | *msrA* | Methionine sulfoxide reductase A; repairs oxidatively damaged methionine residues. | *W. confusa* 119/119 (100.0%); *W. cibaria* 109/109 (100.0%); *W. fermenti* 9/9 (100.0%); *W. tructae* 6/6 (100.0%); *Weissella* sp. 3/5 (60.0%) |
|  | *msrB* | Methionine sulfoxide reductase B; repairs oxidatively damaged methionine residues. | *W. confusa* 119/119 (100.0%); *W. fermenti* 9/9 (100.0%); *Weissella* sp. 2/5 (40.0%) |
|  | *katA/katE* | Catalases; detoxify hydrogen peroxide | *W. confusa* 114/119 (95.8%); *W. viridescens* 11/11 (100.0%); *W. fermenti* 9/9 (100.0%); *Weissella* sp. 1/5 (20.0%) |
| Vitamin biosynthesis/transport | *ribA-H/ribT/ribU/ribZ* | Riboflavin (vitamin B2) biosynthesis or transport-related genes. | *W. confusa* 119/119 (100.0%); *W. cibaria* 109/109 (100.0%); *W. paramesenteroides* 56/56 (100.0%); *W. viridescens* 11/11 (100.0%); *W. fermenti* 9/9 (100.0%); W. soli 8/8 (100.0%); *W. hellenica* 7/7 (100.0%); *W. sagaensis* 6/6 (100.0%); *W. tructae* 6/6 (100.0%); *Weissella* sp. 5/5 (100.0%); *W. jogaejeotgali* 1/1 (100.0%) |
|  | *folA-E/folK/folP/folT* | Folate (vitamin B9) biosynthesis or transport-related genes. | *W. confusa* 119/119 (100.0%); *W. cibaria* 109/109 (100.0%); *W. viridescens* 11/11 (100.0%); *W. fermenti* 9/9 (100.0%); *W. soli* 8/8 (100.0%); W. tructae 6/6 (100.0%); *Weissella* sp. 5/5 (100.0%); *W. minor* 3/3 (100.0%) |
|  | *btuB-D* | Cobalamin/vitamin B12 transport-related genes; do not alone indicate complete B12 biosynthesis. | *W. confusa* 119/119 (100.0%); *W. cibaria* 109/109 (100.0%); *W. paramesenteroides* 56/56 (100.0%); *W. viridescens* 11/11 (100.0%); *W. fermenti* 9/9 (100.0%); *W. soli* 8/8 (100.0%); *W. hellenica* 7/7 (100.0%); *W. sagaensis* 6/6 (100.0%); *Weissella* sp. 5/5 (100.0%); *W. minor* 3/3 (100.0%); *W. uvarum* 2/2 (100.0%); *W. jogaejeotgali* 1/1 (100.0%) |
|  | *thiC-H* | Thiamine (vitamin B1) biosynthesis genes. | *W. confusa* 118/119 (99.2%); *W. cibaria* 109/109 (100.0%); *W. viridescens* 11/11 (100.0%); *W.* fermenti 9/9 (100.0%); *W. hellenica* 7/7 (100.0%); *W. sagaensis* 6/6 (100.0%); *W. tructae* 6/6 (100.0%); *Weissella* sp. 5/5 (100.0%); *W. jogaejeotgali* 1/1 (100.0%) |
|  | *panB-E* | Pantothenate (vitamin B5) biosynthesis genes. | *W. confusa* 6/119 (5.0%); *W. cibaria* 4/109 (3.7%); *Weissella* sp. 1/5 (20.0%) |
